# Receptor-Mediated and Hydrolytic Denitrosylation of Dinitrosyl Iron Complexes to Yield Amorphous Fe_*x*_O_*y*_ and Its Photoinduced Transformation into Crystalline Fe@Fe_*x*_O_*y*_ Nanoparticles

**DOI:** 10.1021/acs.inorgchem.5c01434

**Published:** 2025-06-17

**Authors:** Wun-Yan Wu, Yu-Shen Lin, Linda Iffland, Ulf-Peter Apfel, Tsai-Te Lu, Wen-Feng Liaw

**Affiliations:** † Department of Chemistry, 34881 National Tsing Hua University, Hsinchu 30013, Taiwan; ‡ Department of Chemistry and Biochemistry, Activation of Small Molecules/Technical Electrochemistry, 9142 Ruhr-Universität Bochum, 44801 Bochum, Germany; § Department of Electrosynthesis, Fraunhofer UMSICHT, 46047 Oberhausen, Germany; ∥ Institute of Biomedical Engineering, National Tsing Hua University, Hsinchu 30013, Taiwan

## Abstract

In this study, denitrosylation of amine-bound {Fe­(NO)_2_}^10^ [(2-A)­Fe­(NO)_2_] (**amp-DNIC**) was explored to occur through (a) receptor-mediated transfer of ·NO/[NO]^−^ to [Mn­(TPP)­(Cl)] and (b) a hydrolytic process leading to the liberation of ·NO together with N_2_O. In the presence of the bifunctional ·NO/[NO]^−^-receptor [Mn­(TPP)­(Cl)] (TPP = 5,10,15,20-tetraphenyl-21*H*,23*H*-porphine), **amp-DNIC** acts as a dual ·NO/[NO]^−^ delivery reagent for the conversion of [Mn­(TPP)­(Cl)] into [Mn­(TPP)­(NO)] and [Mn­(TPP)­(NO)_2_]. Alternatively, incubation of **amp-DNIC** in an acetonitrile solution containing 5% deaerated water resulted in its hydrolytic transformation into amorphous Fe_*x*_O_*y*_ particles (**amp-1**) accompanied by the release of ·NO (∼75%), N_2_O (∼10%), and nitrite (∼6%). Upon irradiation of **amp-DNIC** in the presence of the photosensitizer Eosin Y and the sacrificial reductant TEA, the formation of cubic and crystalline Fe@Fe_*x*_O_*y*_ core–shell composite carbonaceous material (**amp-2**) was characterized by high-resolution X-ray diffraction (HRXRD), field emission scanning electron microscopy (FESEM), X-ray photoelectron spectroscopy (XPS), X-ray absorption near-edge spectroscopy (XANES), and extended X-ray absorption fine structure (EXAFS). Based on the mechanisms described above, the hydrolytic transformation of **amp-DNIC** into **amp-1** occurred, followed by the photoinduced structural rearrangement of **amp-1**, amorphous Fe_*x*_O_*y*_ particles, into **amp-2**, the cubic and crystalline Fe@Fe_*x*_O_*y*_ core–shell composite carbonaceous material.

## Graphical abstract

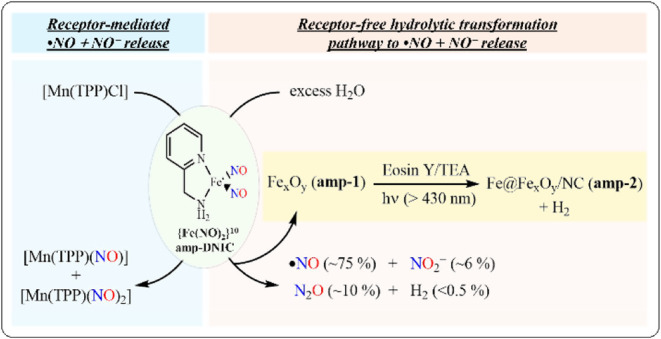

## 1 Introduction

The redox chemistry of nitric oxide (·NO) and its role in various biological processes have been well investigated. To effectively utilize the highly reactive nitric oxide radical while minimizing the immediate production of harmful reactive nitrogen species (RNS), nature has evolved *S*-nitrosothiols (RSNOs) and dinitrosyl iron complexes (DNICs) as storage and transport of ·NO.^1,2^ RSNOs and DNICs are vital for regulating ·NO homeostasis, which is essential for numerous physiological processes. Such processes include vasodilation, relaxation of smooth muscles, prevention of platelet aggregation, memory formation and learning in neurons, and cellular proliferation, differentiation, and apoptosis.^1,2^ HNO/NO^–^ (azanone/nitroxyl), which is a product of the one-electron reduction of nitric oxide, exhibits diverse and unique biological activities in the vascular and myocardial systems.^3–5^ The interconversion between ·NO and HNO/NO^–^ is feasible depending on the redox state of environmental conditions, particularly in the presence of well-known biological reductants such as hydrogen sulfide, alcohols, thiols, and (metallo-)­enzymes.^6,7^ HNO rapidly undergoes dimerization (*k*_HNO_ = 8 × 10^6^ M^–1^ s^–1^) to yield N_2_O and water, restricting its concentration and lifespan in solution. Also, HNO interacts with its counterpart ·NO (*k*_NO,HNO_ = 5.6 × 10^6^ M^–1^ s^–1^) to yield nitrite ion and N_2_O.^6,7^ Thus, the pharmaceutical applications of HNO are typically produced in situ from the rapid decomposition of its thermal donors, including cyanamide, Angeli’s salt, Piloty’s acid, acyl nitroso compounds, and diazeniumdiolate (NONOate).^3–5^ The limitation on biomedical applications of NO^–^/HNO-delivery agents has driven extensive research into the development of controllable metal complex prodrugs, particularly iron nitrosylcomplexes (MNICs).^8–14^ For instance, the light-sensitive {Fe­(NO)}^6^ [Fe­(NO)­(^TMS^PS_2_)­(^TMS^PS_2_H)] produces both ·NO and HNO upon irradiation with a xenon lamp (λ > 400 nm, 150 W), in contrast to the photolysis of the thioether-substituted {Fe­(NO)}^6^ [Fe­(NO)­(^TMS^PS_2_)­(^TMS^PS_2_CH_3_)] which releases only ·NO.^11^

In the field of bioinorganic chemistry, the associated formation of {Fe­(NO)_2_}^9^ DNIC and Roussin’s red ester (RRE) via nitrosylation of nonheme iron proteins/model compounds was established.^15–21^ Among Fe­(NO)_2_}^9^ DNICs, {Fe­(NO)_2_}^9^–{Fe­(NO)_2_}^9^ thiolate-bridged [Fe­(μ-SR)­(NO)_2_]_2_ RREs have been widely utilized to act as NO donors due to the highly covalent nature of the iron-thiolate bonds, which stabilize the nitric oxide ligands.^20,22^ Concerning physiological applications, DNICs and RREs function as efficient ·NO carriers, stabilizing and releasing it within biological systems, exhibiting versatile therapeutic potential in treating hypertension, chronic wounds, viral infections, and various cancers through mechanisms such as vasodilation, angiogenesis, antiviral activity, and tumor suppression.^23–30^ Apoptosis in hepatocellular carcinoma cancer cells through ·NO release from encapsulated {Fe­(NO)_2_}^9^–{Fe­(NO)_2_}^9^ [Fe­(μ-SEt)_2_(NO)_4_] RRE in NanoNO, inhibiting tumor growth, was observed.^31^ Efficiently and appropriate release of the dose of ·NO influences intracellular signaling pathways, promoting the expression of apoptosis-related proteins, which is crucial in cancer therapy.^31^ Recently, mechanistic studies have shown that the intracellular ·NO release of DNICs/RREs for activation of cytoprotective HO-1 can be categorized into two pathways: (a) O_2_-induced decomposition (aerobic condition) and (b) receptor-mediated transfer (anaerobic condition).^32^ Upon ·NO and [NO]^−^ dissociation from DNICs/RREs, the iron released from DNICs and RREs is thought to be stored within ferritin, encapsulating thousands of iron atoms in the form of amorphous ferrihydrite (Fe_*x*_O_*y*_·*n*H_2_O).^33,34^ Beyond serving as an iron reservoir, ferritin interacts dynamically with ·NO, facilitating the formation of low-molecular-weight DNICs/MNICs in mammalian and plant physiology and playing a crucial role in cellular Fe and ·NO homeostasis.^35,36^

Biomimetic investigations into DNICs have yielded unparalleled molecular insights, stemming from advances in synthetic methodologies and electronic structural analyses of a range of mononuclear four/five-coordinate DNICs and binuclear DNICs coordinated/bridged with biologically relevant ligands.^22,37,38^ Specifically, these studies demonstrate that both mononuclear and binuclear DNICs, incorporating RS^–^/R_2_N^–^/RO^–^ donor sets, play crucial roles in dictating the thermodynamic stability/kinetic robustness of [Fe­(NO)_2_]^9/10^ electronic configurations, influencing the oxidation states of NO-coordinated moieties and their chemical reactivity.^22,37^ Advanced spectroscopic methods (XAS, XES, and NRVS) and theoretical calculation suggested the electronic structures of {Fe­(NO)_2_}^9^ as either a high-spin Fe^3+^ (*S* = 5/2) coupling with two triplet [NO]^−^ (S = 1) or a high-spin Fe^2+^ (*S* = 2) coupling with one triplet [NO]^−^ and one ·NO (*S* = 1/2).^22,39,40^ Also, it is known that two NO ligands in the dinitrosyl iron unit (DNIU) trigger intramolecular ON–NO coupling, which is unfavorable due to the strong antiferromagnetic interaction between the iron center and NO moieties, leading to the mutual spin-parallel alignment of the two NO.^39,40^ The lower effective nuclear charge (*Z*_eff_) of Fe^2+^ causes the metal 3d orbitals to be closer in energy to the π* orbitals, resulting in more pronounced π-back-bonding, and the electronic structures of {Fe­(NO)_2_}^10^ is then assigned as [Fe^II^(NO^–^)­(NO^–^)]. Additionally, “hard” alkoxide/phenolate ligands tend to polarize the {Fe­(NO)_2_}^9^ core to cause more [Fe^III^(NO^–^)­(NO^–^)]^−^ character compared to the “soft” *S*-ligating DNICs.^41^ Very recently, a fully delocalized dinuclear DNIC [Fe­(NO)_2_(μ-ON_2_^Me^)­Fe­(NO)_2_] is demonstrated to be endowed with the spin-frustrated {Fe­(NO)_2_}^9^–{Fe­(NO)_2_}^9^–[·ON_2_^Me^]^−2^ electron configuration (*S*_total_ = 1/2) with the “hard” *O*-phenoxide moiety polarizing the iron center(s) of [Fe­(NO)_2_] unit(s) to enforce the “constrained” π-conjugation imino-substituted phenolate system.^42^ However, how ligands and the oxidation state in DNICs/RREs regulate the release of nitric oxide and its derivatives currently remains elusive. Although the water-tolerant {Fe­(NO)_2_}^9^–{Fe­(NO)_2_}^9^ [Fe­(μ-SR)­(NO)_2_]_2_ RREs have been recognized as controlled ·NO donors, activated by photo irradiation, oxygen, and receptor-mediated ·NO transportation (e.g., deoxyHb and metalloporphyrin),^43^ the dinuclear {Fe­(NO)_2_}^9^–{Fe­(NO)_2_}^9^ [Fe­(μ-^Me^Pyr)­(NO)_2_]_2_ DNIC has been revealed as a potential nitroxyl-releasing reagent mediated by Fe-porphyrin centers (deoxyHb/metMb/Fe­(TPP)­Cl, TPP = tetraphenylporphyrin).^43^

## 2 Results and Discussion

### 2.1 Receptor [Mn­(TPP)­(Cl)]-Mediated ·NO + [NO]^−^ Transfer from Nitrogen-Bound {Fe­(NO)_2_}^10^[(2-A)­Fe­(NO)_2_] DNIC (**amp-DNIC**)

In contrast to the widely investigated {Fe­(NO)_2_}^9^ DNICs/RREs acting as a NO/NO^–^ releasing regent, {Fe­(NO)_2_}^10^ DNICs are rarely studied and adopted to serve as a NO/NO^–^ releasing regent, due to their highly reductive reactivity. Recently, the amine-bound mononuclear {Fe­(NO)_2_}^10^ [(2-A)­Fe­(NO)_2_] (2-A = 2-aminomethylpyridine) was reported to serve as a precursor for converting exogenous NO to HNO/N_2_O.^37^ Preparation of {Fe­(NO)_2_}^10^ [(2-A)­Fe­(NO)_2_] (**amp-DNIC**) was followed by the previous work.^37^ Addition of 1.0 equiv of Fe­(CO)_2_(NO)_2_ into the THF solution of 2-A yielded {Fe­(NO)_2_}^10^ **amp-DNIC**. IR ν_NO_ stretching frequencies (1694, 1642 cm^–1^) of **amp-DNIC** were known as the feature of mononuclear {Fe­(NO)_2_}^10^ DNIC.^57^ The Fe Mössbauer spectrum of {Fe­(NO)_2_}^10^ **amp-DNIC** displays the isomer shift (δ) of 0.25 mm/s, which falls within the isomer shift range of δ = 0.19–0.37 mm/s for π-/σ-donating nitrogen-bound {Fe­(NO)_2_}^9/10^ DNICs (Figure S1).^37^ It is noticed that when THF solution of [Mn­(TPP)­(Cl)] was added with 1.0 equiv of **amp-DNIC**, and an immediate change in solution color from light green to brown was observed at ambient temperature. The UV–vis spectrum shows that [Mn­(TPP)­(Cl)] (557 and 623 nm (THF)) was immediately converted into [Mn­(TPP)­(NO)] (570 and 605 nm (THF)) via NO^–^ group transfer from **amp-DNIC** (Scheme 1a and Figure 1a).^44,45^ Cyclic voltammograms (CVs) of **amp-DNIC** and [Mn­(TPP)­(Cl)] were investigated in THF (0.8–1.2 mM) with 0.1 M [*n*-Bu_4_N]­[PF_6_] as the supporting electrolyte. As shown in Figure 2, **amp-DNIC** and [Mn­(TPP)­(Cl)], individually, display reversible redox couples at *E*_1/2_ = −0.52 V (*E*_pa_ = −0.48 V, *E*_pc_ = −0.56 V, Δ*E*_p_ = 0.08 V, *i*_pa_/*i*_pc_ = 1.01 vs Fc/Fc^+^) for **amp-DNIC** shuttling between {Fe­(NO)_2_}^10^ ↔ {Fe­(NO)_2_}^9^, and at *E*_1/2_ = −0.64 V (*E*_pa_ = −0.60 V, *E*_pc_ = −0.67 V, Δ*E*_p_ = 0.07 V, *i*_pa_/*i*_pc_ = 1.02 vs Fc/Fc^+^) for [Mn^III^(TPP)­(Cl)] shuttling between Mn^III^ and Mn^II^. These redox couples of **amp-DNIC** and [Mn­(TPP)­(Cl)] demonstrate that **amp-DNIC** is not able to reduce Mn^III^ to Mn^II^ in the reaction of **amp-DNIC** and [Mn­(TPP)­Cl]. On the contrary, a direct [NO]^−^ group transfers from **amp-DNIC** to [Mn­(TPP)­Cl] to generate [Mn­(TPP)­(NO)]. Subsequently, the resulting brownish solution of [Mn­(TPP)­(NO)] was gradually converted to red solution after 1 h, consistent with the formation of [Mn­(TPP)­(NO)_2_] (UV–vis spectra 540 and 575 nm, as displayed in Scheme 1b and Figure 1a). In a similar fashion, FTIR spectra also demonstrate the rapid consumption of a half equivalent of **amp-DNIC** when THF solution of [Mn^III^(TPP)­Cl] was added to **amp-DNIC**, resulting in the appearance of peak at 1742 cm^–1^ (assigned as [Mn­(TPP)­(NO)]) along with the stretching frequency at 1750 cm^–1^ (assigned as [Mn­(TPP)­(NO)_2_]), as shown in Figure 1b.^45^ The stretching frequency at 1750 cm^–1^ becomes dominant after the reaction solution was stirred for 90 min (Scheme 1a), implicating **amp-DNIC** transfers [NO]^−^ to [Mn^III^(TPP)­Cl] followed by the subsequent transfer of ·NO to [Mn­(TPP)­(NO)] yielding [Mn­(TPP)­(NO)_2_] (Scheme 1b).^45^ These results are consistent with the observation that the nitrosylation of manganese-porphyrin complex [Mn^II^(TPP)] with ·NO rapidly yielded {Mn­(NO)}^6^ [Mn­(TPP)­(NO)], which subsequently reacted with excess ·NO to generate the {Mn­(NO)_2_}^7^ [Mn­(TPP)­(NO)_2_] complex.^44–48^ The commercially available ·NO-trapping reagent [Co­(TPP)] (Co­(TPP) = 5,10,15,20-tetraphenyl-21*H*,23*H*-porphine cobalt­(II)) and [NO]^−^-trapping reagent [Mn­(TPP)­Cl] (Mn­(TPP)Cl = 5,10,15,20-tetraphenyl-21*H*,23*H*-porphine manganese­(III) chloride) were employed for mediating the denitrosylation of **amp-DNIC**.^44,45^ FTIR spectra indicate that [Co­(TPP)] does not induce NO transfer reaction upon adding two equivalents of Co­(TPP) into the THF solution of **amp-DNIC** under a nitrogen atmosphere (Figure S2). These results indicate that [Co^II^(TPP)] does not induce the release of ·NO of **amp-DNIC**. Instead, [Mn­(TPP)­Cl] does induce [NO]^−^ transfer from **amp-DNIC** and subsequently promotes the liberation of ·NO. It is proposed that the [NO]^−^ moiety released from **amp-DNIC** is transferred to [Mn^III^(TPP)]^+^, forming {Mn­(NO)}^6^ [Mn­(TPP)­(NO)] along with the {Fe­(NO)}^8^ intermediate. Subsequently, due to its intrinsic instability or mediation by the {Mn­(NO)}^6^ [Mn­(TPP)­(NO)], the {Fe­(NO)}^8^ intermediate facilitates the liberation of ·NO bound to [Mn­(TPP)­(NO)], leading to the formation of {Mn­(NO)_2_}^7^ [Mn­(TPP)­(NO)_2_] (Scheme 1a,b).^44^ In summary, although most mono- and dinuclear MNICs^49–53^ as well as diiron metalloproteins (e.g. YtfE)^54^ tend to rearrange into {Fe­(NO)_2_}^9^ DNIC or {Fe­(NO)_2_}^10^ DNIC via redox reactions or excessive ·NO exposure, in this study, we uncover a receptor-mediated pathway to denitrosylate {Fe­(NO)_2_}^10^ species.

**Scheme 1:**
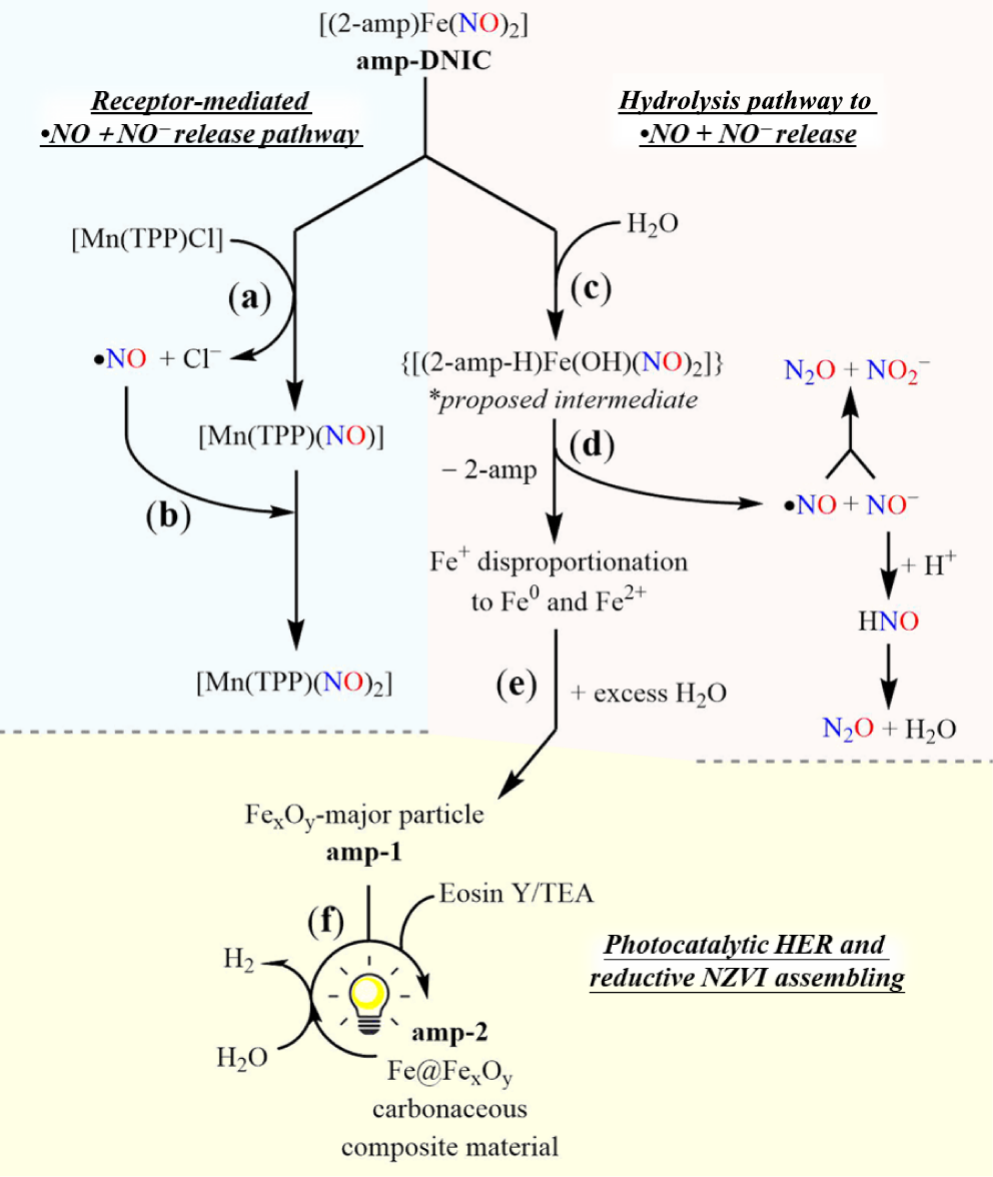
Scheme for the Proposed Mechanism^a^

a The blue region shows the receptor-mediated pathway to generate [Mn­(TPP)­(NO)]_2_. The red region displays the hydrolysis pathway forming **amp-1**. The yellow region delineates the three-component photocatalytic HER and the formation of **amp-2** NZVI. (a) [Mn­(TPP)­Cl]-mediated nitroxyl transfer from **amp-DNIC**, yielding [Mn­(TPP)­(NO)]. (b) The subsequent liberation of NO, followed by the interaction of NO and [Mn­(TPP)­(NO)], yielding [Mn­(TPP)­(NO)_2_]. (c) Hydrolysis of {Fe­(NO)_2_}^10^ **amp-DNIC.** (d) The proposed Fe^+^ disproportionation yielding Fe^0^/Fe^2+^ and the released ·NO + NO^–^ interaction to produce N_2_O + [NO_2_]^−^ along with the concomitant HNO dimerization yielding N_2_O + H_2_O. (e) Proposed iron species passivation and aggregation, yielding **amp-1**. (f) Photocatalytic HER and reductive assembling of **amp-2** NZVI

**Figure 1:**
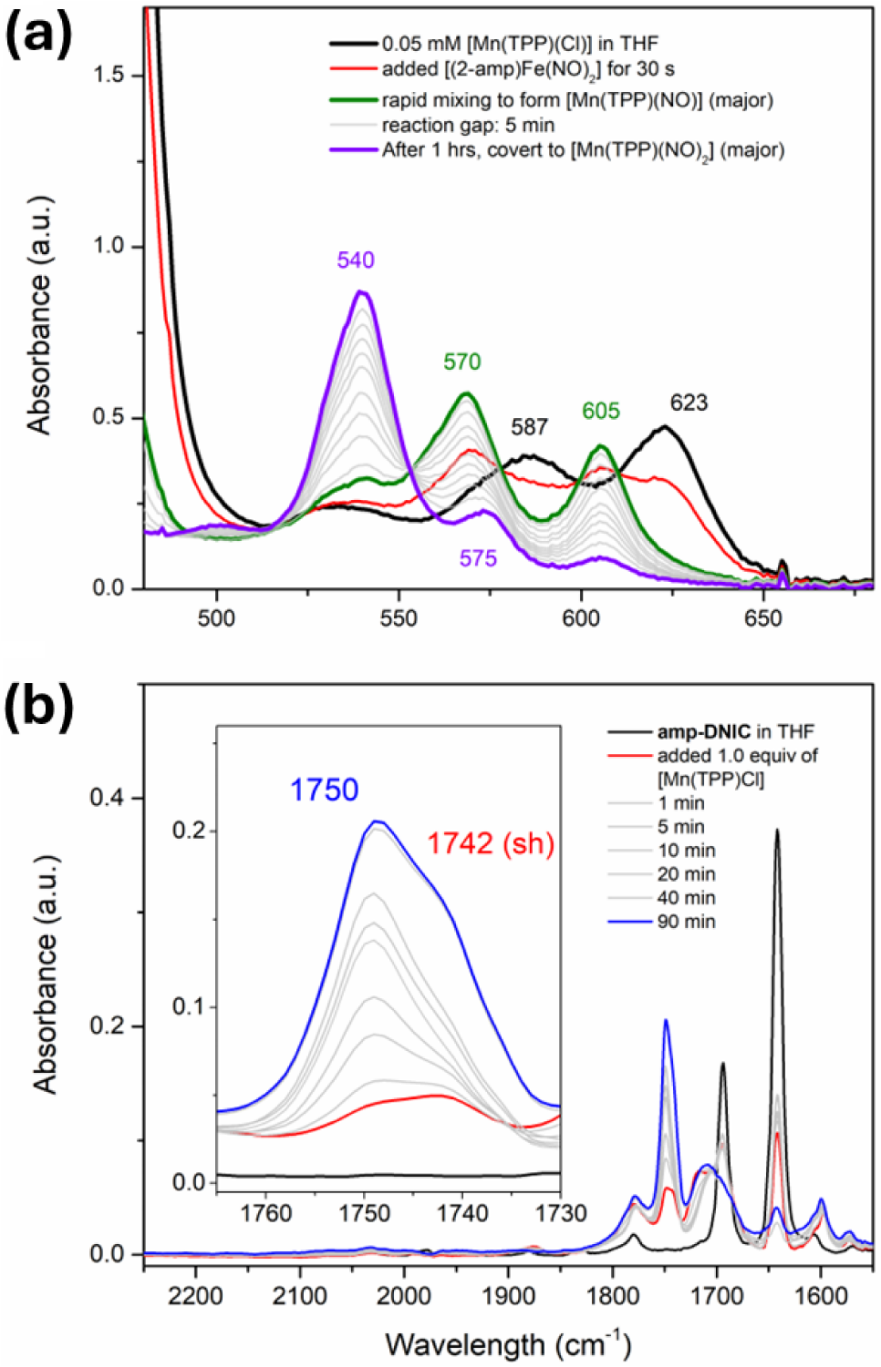
(a) Black line: UV–vis spectrum of [Mn­(TPP)­(Cl)]. Green line: UV–vis spectra were obtained from reaction of **amp-DNIC** and [Mn­(TPP)­(Cl)] (1:1 molar ratio) in THF solution. Purple line: UV–vis spectrum obtained from the reaction mixture of **amp-DNIC** and [Mn­(TPP)­(Cl)] stirred for 1 h yielding [Mn­(TPP)­(NO)_2_] (540 nm). (b) FTIR spectra (THF) show the stretching frequencies 1742 cm^–1^ (assigned as [Mn­(TPP)­(NO)]) and 1750 cm^–1^ (assigned as [Mn­(TPP)­(NO)_2_]) upon mixing THF solution of **amp-DNIC** and [Mn­(TPP)­(Cl)] (1:1 molar ratio), and the stretching frequency at 1750 cm^–1^ (assigned as [Mn­(TPP)­(NO)_2_]) grows after the mixture solution was stirred for 90 min.

**Figure 2:**
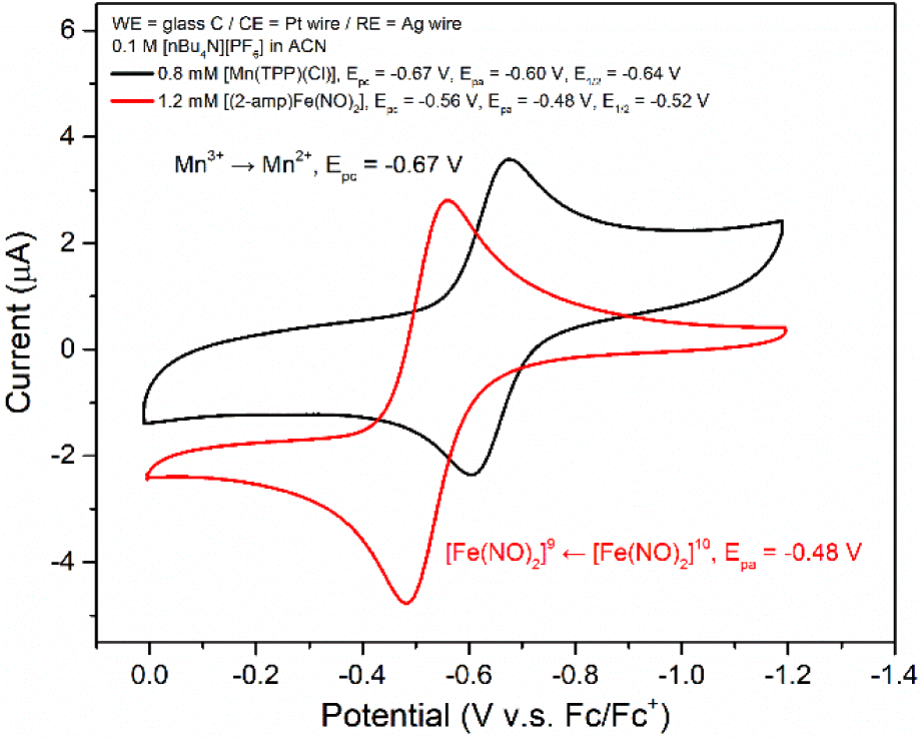
Cyclic voltammetry of 0.8 mM [Mn­(TPP)­(Cl)] (black) and 1.2 mM **amp-DNIC** (red line), individually, measured in a THF solution with 0.1 M [*n*-Bu_4_N]­[PF_6_] as the supporting electrolyte (ferrocene as the internal standard at room temperature; scan rate, 0.02 V/s).

### 2.2 Hydrolysis of **amp-DNIC** Liberating ·NO + NO^–^ Accompanied by Formation of Iron-Based Particles

As known, {Fe­(NO)_2_}^10^ DNICs proceed via facile oxidation to yield {Fe­(NO)_2_}^9^ DNICs along with hydrogen liberation in the presence of moderate acid source.^55^ Recently, we demonstrated that the nitrogen-bound {Fe­(NO)_2_}^10^ [(L)­Fe­(NO)_2_] (L = Me_6_tren, PMDTA, ^Me^DDB, N_3_MDA) DNICs act as precursors of Fe@Fe_*x*_O_*y*_ nanoparticles under the photocatalytic process.^56–58^ The one-electron oxidative transformation of dinuclear {Fe­(NO)_2_}^10^–{Fe­(NO)_2_}^10^ [Fe_2_(^Pyrr^PDI)­(NO)_4_] DNIC to dinuclear [{Fe­(NO)_2_}_2_]^9/10^ DNIC [Fe_2_(^Pyrr^PDI)­(NO)_4_]^+^ in acetonitrile triggers the site lability of the electron-deficient {Fe­(NO)_2_}^10^ motif to undergo intra/intermolecular N–N bond formation, leading to the formation of {Fe­(NO)_2_}^9^ DNIC [Fe­(^Pyrr^PDI)­(NO)_2_]^+^ along with N_2_O and Fe_*x*_O_*y*_.^59^ Very recently, the presence of the secondary sphere coordination pendant anilines in {Fe­(NO)_2_}^10^ Fe­(^PhNH^PDI)­(NO)_2_ DNIC enhances the hydrogen bonding capabilities, facilitating N–N coupling to form N_2_O and Fe_*x*_O_*y*_.^60^ Drawing inspiration from the transformation of [Fe­(NO)_2_]^10^ DNICs into iron-based particles in H_2_O-triggered conversion, the stability study of **amp-DNIC** in the presence of water was conducted (Scheme 1c). As shown in Figure 3a–c, the freshly prepared acetonitrile solution of **amp-DNIC** (IR_NO_ stretching frequency 1635 and 1689 cm^–1^) added with 5% deaerated water was gradually converted to brownish colloid solution with the appearance of 2228 cm^–1^ IR_NO_ stretching frequency (N_2_O) and the simultaneous generation of ·NO and N_2_O characterized by GC-BID. The yield of released N_2_O was quantitated as 10.8 ± 0.8%, corrected by the calibration curve (Scheme 2 and Figure S3), implying that the redox event occurred in DNIU mainly leading to ·NO + N_2_O release. Also, the trace amounts of H_2_ (<0.5%) were detected (Figure 3c). Upon hydrolysis of **amp-DNIC** in the presence of 5% deaerated water, the tube-to-cell trapping the released ·NO by [Co­(TPP)] was conducted to lead to the isolation of the {CoNO}^8^ [Co­(TPP)­(NO)] (TPP = 5,10,15,20-tetraphenyl-21H,23H-porphine), as displayed in Figure S4.^61,62^ The ·NO trapping monitored by UV–vis spectra was analyzed using linear combination fitting (LCF) with reference spectra of [Co­(TPP)] and [Co­(TPP)­(NO)]. The LCF analysis suggested that the liberation of ·NO from **amp-DNIC** was calculated to be approximately 75.8 ± 3.7% (Figure S5). The yield of nitrite ion was extracted by deaerated water after centrifugation and was quantified via Griess assay (6.1 ± 0.4%, Figure S6). We noticed that the addition of triethylamine or NaOH to an acetonitrile solution containing 5% water dramatically accelerates the decomposition of **amp-DNIC**. Reaction of **amp-DNIC** and 1 equiv of tetrabutylammonium hydroxide (TBAOH) in deaerated and anhydrous acetonitrile led to the formation of insoluble solid. FTIR analysis revealed the decrease of the characteristic IR_NO_ stretching frequencies 1635 and 1689 cm^–1^ (**amp-DNIC**) within 5 min, followed by the gradual emergence of new absorption bands at 1708 and 1682 cm^–1^ after 30 min (Figure S7a). These newly formed bands, consistent with those observed for acetonitrile solution of TBAOH, suggest the hydroxide-driven hydrolysis of acetonitrile (Figure S7b).^63^ Low temperature UV–vis spectra indicate that the reaction between **amp-DNIC** and one equivalent of TBAOH leads to an overall elevation of the spectral intensity (Figure S8). These results suggest that hydroxide ion-mediated degradation of **amp-DNIC** yields insoluble suspended particles with no intermediates detected at −40 °C. Upon warming the reaction solution to room temperature and leaving it to stand for 3 h, a significant increase of band intensity of 400 nm was observed (Figure S9a). This observation is consistent with the hydroxide-driven hydrolysis reaction in acetonitrile containing only TBAOH (Figure S9b).^63^ In addition, when the solvent was replaced with THF, the reaction of **amp-DNIC** and TBAOH in THF resulted in a rapid decrease in the IR_NO_ stretching frequency at 1635 and 1689 cm^–1^ (**amp-DNIC**) (Figure S10), consistent with the reaction observed in acetonitrile. It is presumed that the 2-A ligand may trigger the deprotonation of water, which promotes the generation of hydroxide ions to be bound to the oxyphilic iron center.^64,65^ The binding of hydroxide ions may increase the ionic character of the Fe-NO bond, facilitating the denitrosylation of DNIU to release ·NO + [NO]^−^ and ultimately leading to the formation of iron-based products (Scheme 1c,d).^41^

**Figure 3:**
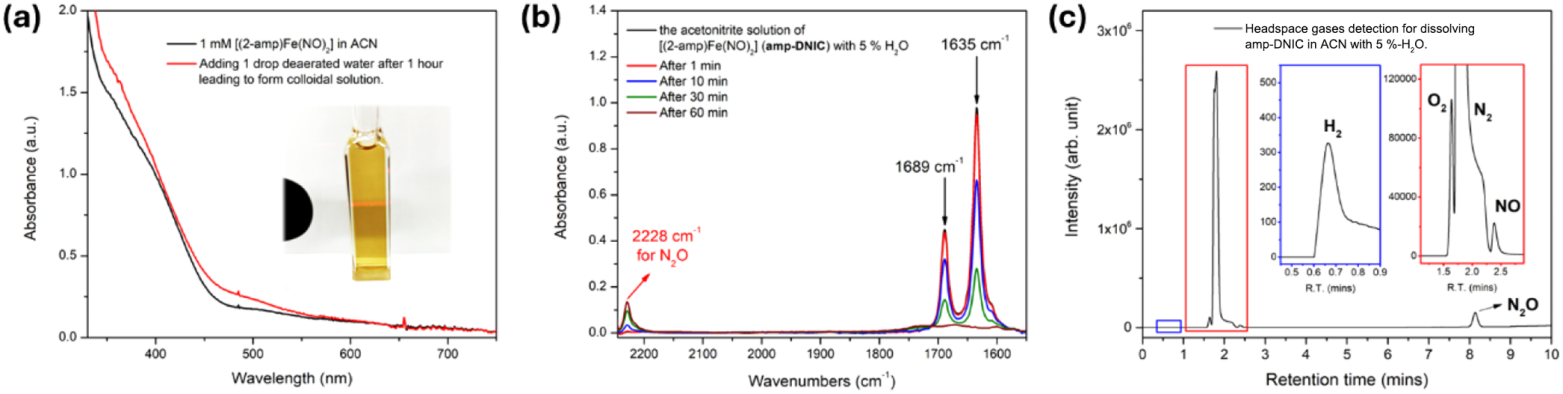
(a) UV–vis spectra of 1 mM **amp-DNIC** in acetonitrile (black) and then the CH_3_CN solution of **amp-DNIC** reacted with 1 drop of deaerated water for 1 h (red line). (b) FTIR spectra of the acetonitrile solution of **amp-DNIC** containing 5% H_2_O stirred for 1 h and monitored (peak intensities at 1689 and 1635 cm^–1^ decrease with the formation of N_2_O (2228 cm^–1^)). (c) Headspace gases detected by GC from the hydrolysis of **amp-DNIC** (Inset: H_2_ (blue region) and O_2_/N_2_/NO (red region)).

**Scheme 2:**
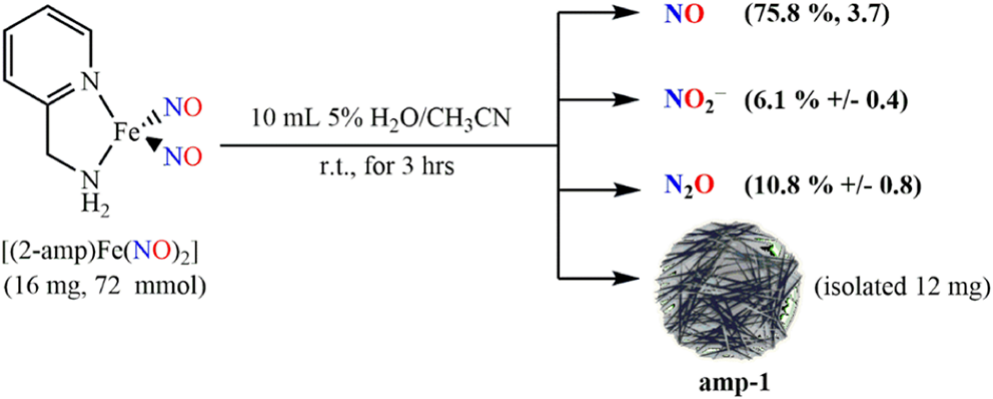
Transformation of {Fe­(NO)_2_}^10^ [(2-amp)­Fe­(NO)_2_] DNIC to ·NO + NO_2_^–^ + N_2_O and Iron Oxide-Based **amp-1** in the Acetonitrile Solution Containing 5% Deaerated Water[Fn sch2-fn2]

a The yield distribution of ·NO, NO_2_^–^, and N_2_O, individually, was analyzed by GC-BID and Griess test, and the details are presented in Section 4.

The resulting colloidal suspension is isolated as brown powder **amp-1** (12 mg) via centrifugation (Scheme 1). ICP-MS is used to analyze the conversion of **amp-DNIC** to **amp-1**, with a conversion rate of 74.1% based on the moles of Fe. The morphology of **amp-1** is analyzed using field emission scanning electron microscopy (FESEM). The structure of nanoscale **amp-1** is roughly flocculent, with dimensions ranging from approximately 91 to 102 nm (Figure 4a,b). The EDX mapping of **amp-1** indicates that **amp-1** is primarily composed of C (16.74%), O (52.20%), and Fe (28.51%) with smaller amounts of N (2.55%), as displayed in Figure S11. High-resolution powder X-ray diffraction (radiation source energy set to 20 keV, λ = 0.61992 Å) of **amp-1** presents an amorphous pattern without any perceptible peaks ranging from 15.0° to 45.0° (Figure 5). X-ray photoelectron spectroscopy (XPS) spectra of **amp-1**, the depth profile with increasing etching depth and with respect to an Fe foil reference, show the increasing component of the Fe element (from 19.9% to 46.5%) and the decreasing components of C (from 32.0% to 15.3%) and O (from 44.2% to 34.8%) with an organic layer covering the surface (Figure 6a). The EDX and XPS atomic ratio corresponding to organic species deposition (2-A ligand and derivatives) on the surface of **amp-1** was demonstrated by solid-state FTIR (Figure S12). The Fe 2p XPS depth profile (DP) of **amp-1** reveals the increasing component of metallic iron (binding energy at ∼706 eV) from the surface to the inner layer (Figure 6b). The inner layer fitting indicates that the percentage composition of Fe^0^, Fe^2+^, and Fe^3+^ is 5.4%, 65.0%, and 30.6%, respectively (Figure 6c). To avoid overestimating the Fe^0^ composition in **amp-1**, the Fe 2p XPS depth profile of Fe_3_O_4_ reveals only a trace formation of Fe^0^ (Figure S13). Prolonged X-ray irradiation (XPS depth profile experiment) causes **amp-1** decomposition.^66–68^ In addition, the Fe K-edge X-ray absorption near edge structure (XANES) spectrum of **amp-1** (Fe foil, FeO, Fe_2_O_3_, and Fe_3_O_4_ as the references) supports the percentage composition (3.0 ± 1.0%) of metallic iron (Figures 6d and 7a). To reveal the detailed chemical state and coordination environment of **amp-1**, Fe K-edge Fourier transform extended X-ray absorption fine structure spectroscopy (EXAFS) analysis was conducted to reveal the Fe–O bond distance (1.53 Å) and Fe···Fe distance (2.62 Å), with oscillation functions *k* ^3^χ­(*k*) ranging from 0 to 12 Å^–1^ and uneven structural uniformity (Figure 7). As displayed in Figure S14, the Mössbauer spectrum of **amp-1** reveals two distinct sites: (a) isomer shifts (δ) of 0.43 mm/s with quadrupole splitting (Δ*E*_Q_) of 0.88 mm/s (major site) and (b) isomer shifts (δ) of 0.45 mm/s with quadrupole splitting (Δ*E*_Q_) of 0.47 mm/s (minor site). These values are comparable to those of amorphous iron oxides (Fe_*x*_O_*y*_; δ = 0.34–0.48 mm/s and Δ*E*_Q_ = 0.32–0.84 mm/s),^59,60,69^ which were prepared through the thermolysis of Prussian Blue and redox-triggered denitrosylation of dinuclear {Fe­(NO)_2_}^10^–{Fe­(NO)_2_}^10^ DNIC. Additionally, some possible magnetic species referring to standards may be covered in the noise of the fingerprint baseline (Figure S10 and Table S1), implying the formation of in situ low-valent iron species and its passivation under hydrolytic denitrosylation. Taken together, on the basis of PXRD, XPS, and XANES/EXAFS analyses, the major component of **amp-1** should be defined as an amorphous Fe_*x*_O_*y*_-type species.

**Figure 4:**
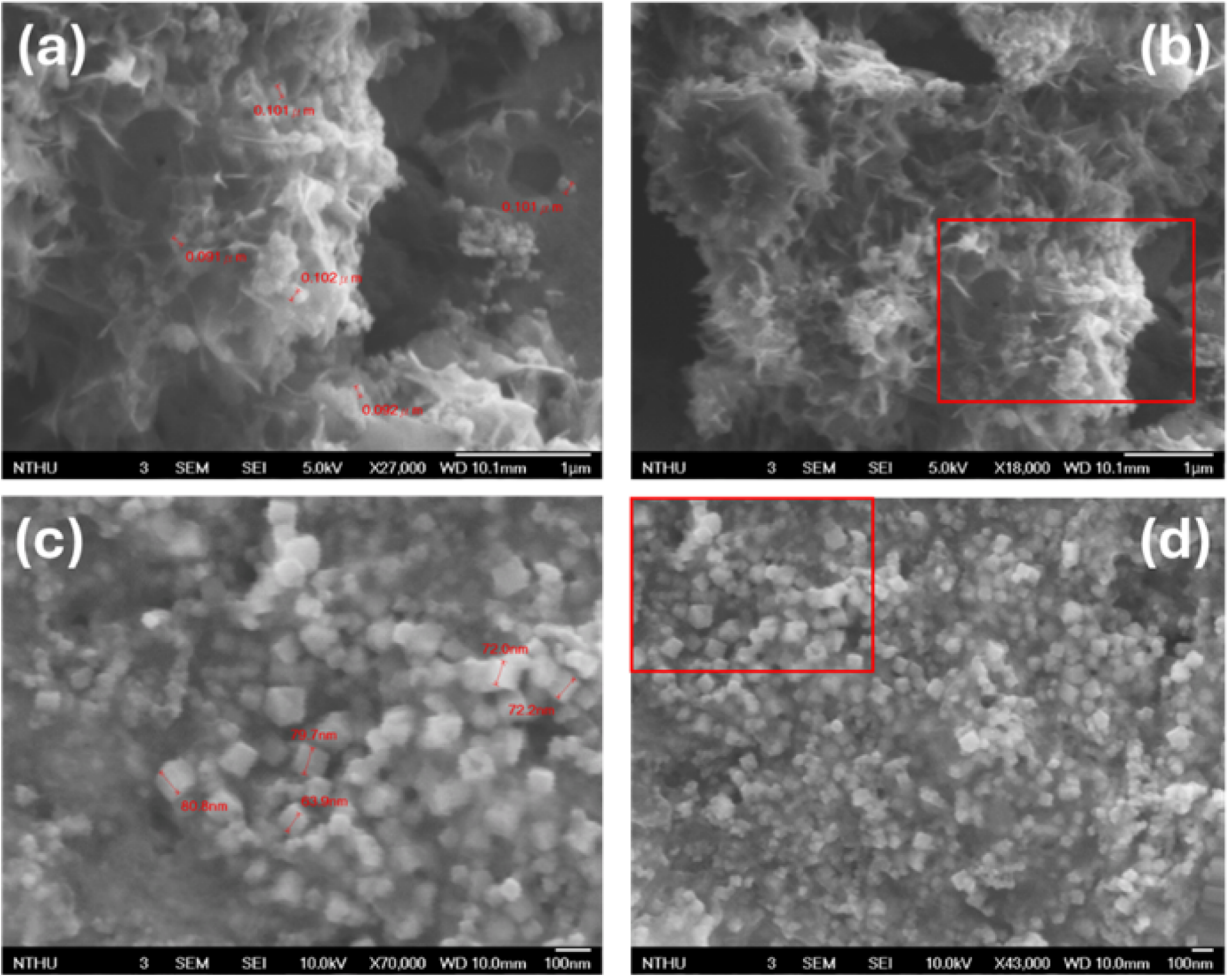
FESEM images of (a, b) **amp-1** (97.4 nm ± 5.4) and (c, d) **amp-2** (73.7 nm ± 5.2).

**Figure 5:**
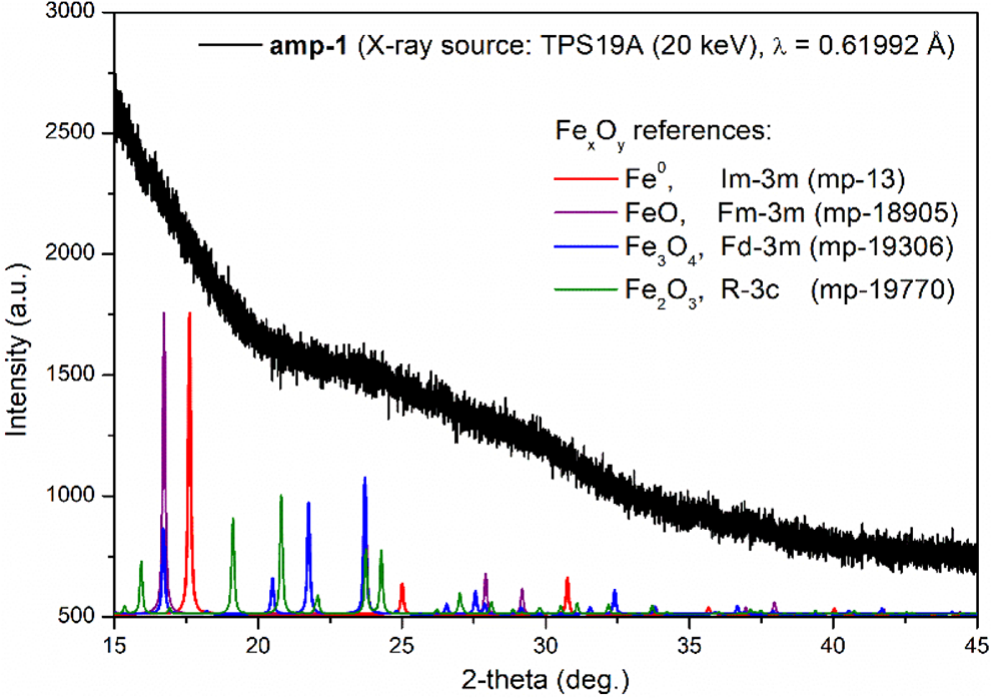
Powder diffraction pattern of **amp-1**, zero valence iron (*Im*3̅*m*), Fe_3_O_4_ (*Fd*3̅*m*), Fe_2_O_3_ (*R*3̅*c*), and FeO (*Fm*3̅*m*) databases. Experimental data collections were performed by high-resolution X-ray diffraction (energy set to 20 keV (λ = 0.61992 Å), TPS 19A, NSRRC, Taiwan).

**Figure 6:**
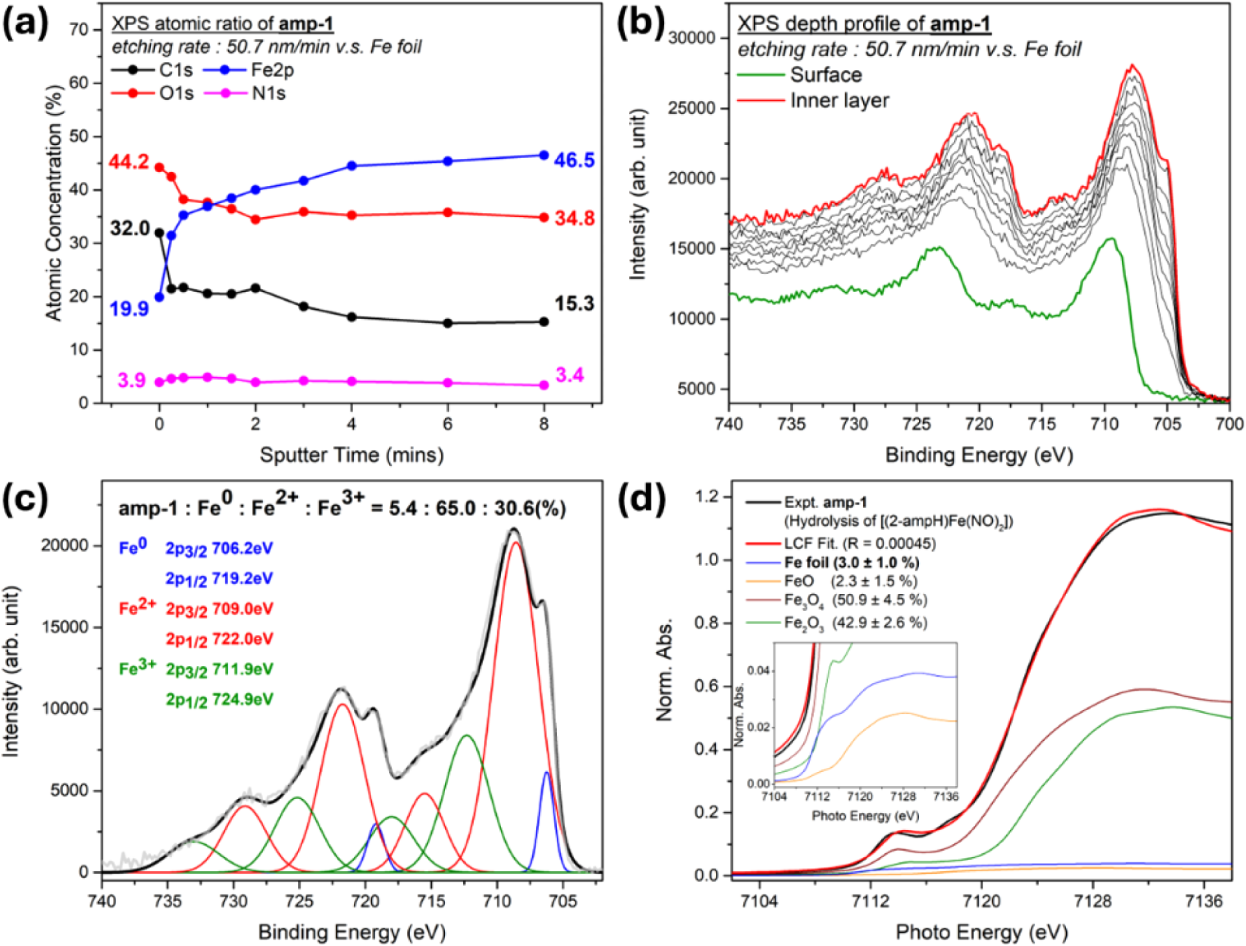
(a) XPS depth profile of the atomic ratio for **amp-1** (etch rate: 50.7 nm/min versus Fe foil). (b) XPS Fe 2p spectra for **amp-1** at different etch depths. (c) XPS Fe 2p fitting for the inner layer of **amp-1**. (d) Fe K edge preedge XAS spectra of **amp-1** (black line) and the linear combination fitting of Fe foil, FeO, Fe_3_O_4_, and Fe_2_O_3_ (red line).

**Figure 7:**
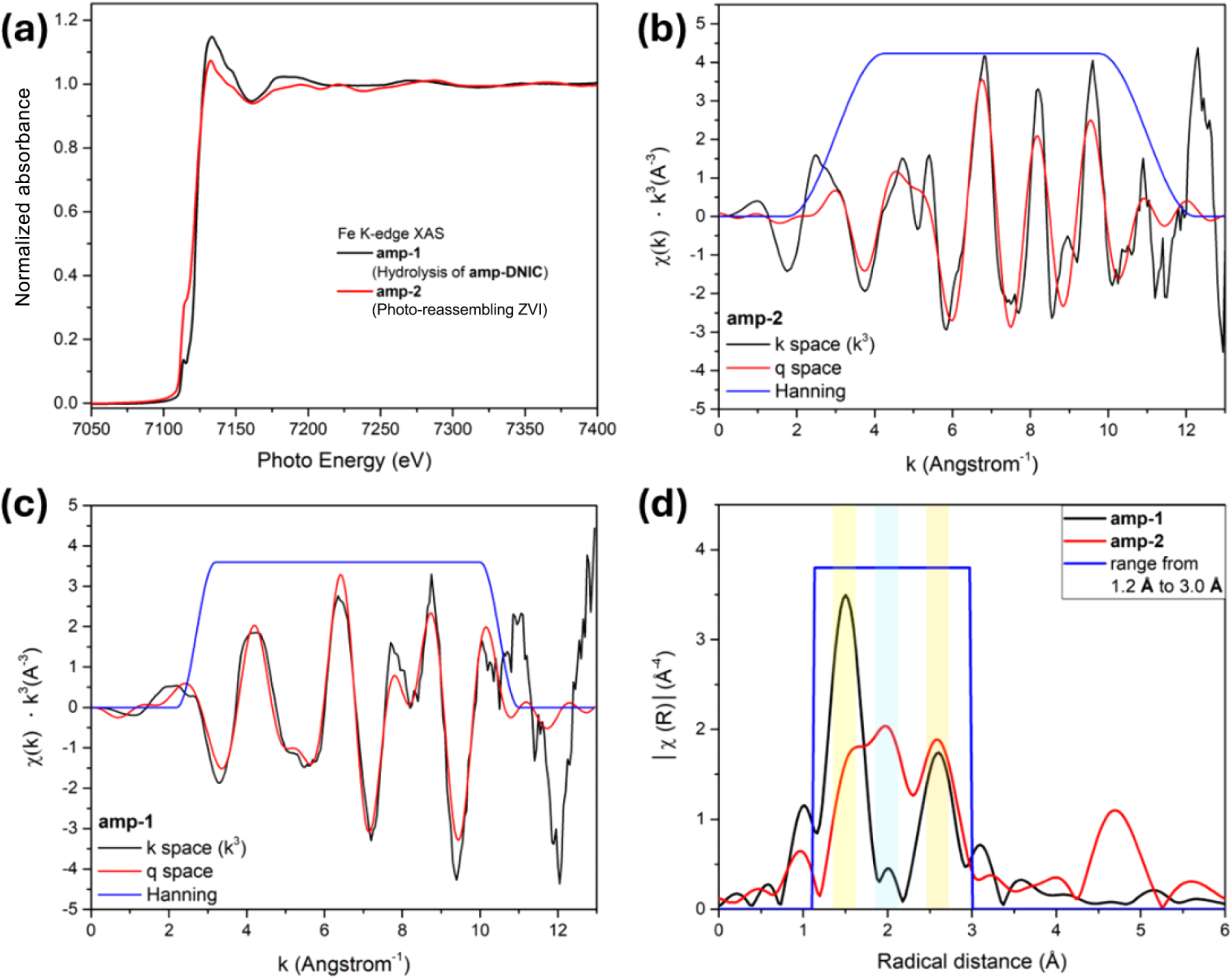
(a) Fe K-edge XANES spectra of **amp-1** (black line) and **amp-2** (red line). Fe K-edge EXAFS oscillation functions *k* ^3^χ­(*k*) and *q*-space (based on *R*-space ranging from 1.2 to 3.0 Å) of (b) **amp-1** and (c) **amp-2**. (d) FT-EXAFS curves (*R* space) at the Fe K-edge of **amp-1** (black) and **amp-2** (red) (Fe–O bond distance ∼ 1.53 Å (yellow zoom) for Fe_*x*_O_*y*_; Fe–Fe bond distance ∼ 2.01 Å (blue zoom) for metallic Fe; Fe···Fe distance ∼ 2.62 Å (orange zoom) for Fe_*x*_O_*y*_).

It is proposed that the water-triggered transformation of **amp-DNIC** to **amp-1** may occur through the hydroxide-bound intermediate [(2-A-**H** ^**+**^)­Fe­(^−^ **OH**)­(NO)_2_], which polarizes the Fe-NO bond of the DNIU to result in the synergistic heterolytic-homolytic cleavage of the Fe-NO bond, generating ·NO + NO^–^ and Fe^+^ species (Schemes 1c,d). The released ·NO and NO^–^ rapidly undergo interaction (2 ·NO + NO^–^ → NO_2_^–^ + N_2_O and 2 HNO → N_2_O + H_2_O) to result in the formation of N_2_O + nitrite (Scheme 1d). The trace amount of Fe^0^ may be rationalized by the disproportionation of Fe^+^ into Fe^2+^ and Fe^0^ species, prior to aggregation and passivation forming Fe_*x*_O_*y*_-major **amp-1** (Schemes 1e). Also, it is proposed that neutral ·NO molecules adsorb onto Fe_*x*_O_*y*_ less effectively than anionic [NO]^−^, and iron oxides exhibit strong oxophilicity, making their surfaces favor adsorbing water (H_2_O, OH^–^) rather than neutral ·NO. Electrochemical and theoretical calculations of the redox potential of nitric oxide and iron species in water suggest that the released [NO]^−^ species exhibits stronger reductive potential with reduction potentials of *E*_NO‑to‑[NO]^−^_ = −0.68 V (at pH 7) and −0.79 V (at pH 13) versus SHE.^70^ This implies that the liberated [NO]^−^ may partially reduce Fe_*x*_O_*y*_ to form iron species along with the major product ·NO, compared to the reduction potential −0.44 V/–0.51 V versus SHE (at pH 7) for Fe^2+^/Fe­(OH)_2_ reduced to Fe^0^.^71^

### 2.3 Photoinduced Reduction of **amp-1** in the Presence of Eosin Y + TEA Leading to Fe^0^@Fe_*x*_O_*y*_ NPs along with H_2_ Evolution

In the previous study, the nitrogen-bound {Fe­(NO)_2_}^10^ [(N_3_MDA)­Fe­(NO)_2_] DNIC was employed to work in concert with a xanthene photosensitizer and a sacrificial reductant (TEA), presenting a one-pot photosynthetic method for synthesizing well-dispersed cubic Fe@Fe_3_O_4_ core–shell nanoparticles embedded in an N-doped carbonaceous polymer (NZVI@NC). The transformation of DNIC to Fe@Fe_3_O_4_ core–shell nanoparticles may involve the reductive denitrification of {Fe­(NO)_2_}^10^ [(N_3_MDA)­Fe­(NO)_2_] DNIC, followed by subsequent reassembly into zerovalent iron species along with releasing N_2_O.^72^ As mentioned above, the lower stability in excess deaerated water triggered the hydrolytic transformation of **amp-DNIC** to **amp-1**, accompanied by the liberation of ·NO and N_2_O. Consequently, the role of the DNIC precursor in the preparation of NZVI particles should be reconsidered and further elaborated. In a similar approach, the conversion of **amp-1** into Fe^0^@Fe_*x*_O_*y*_ NPs in the presence of Eosin Y + TEA was also achieved via photoinduced reductive assembly. It is noted that {Fe­(NO)_2_}^10^ **amp-DNIC** shows a certain degree of photodurability in THF (Scheme 1f and Figure S15). As shown in Scheme 3, the freshly prepared acetonitrile solution (9.5 mL) of **amp-DNIC** in a 200 mL photoreactor equipped with a Graham condenser, with the addition of 0.5 mL deaerated water and subsequent stirring for 1 h, led to the brownish colloid solution of **amp-1**. The freshly prepared deaerated water solution of Eosin Y and TEA was added to the brownish colloid solution of **amp-1** and then left to stand for 30 min in the dark to measure the headspace gas by GC-BID, as shown in Figure 8a,c. The mixture solution containing **amp-1**, Eosin Y and TEA was irradiated with a 20 W LED (λ > 420 nm) for 24 h at ambient temperature, resulting in the formation of a fluorescent yellow solution, along with suspended black powder (**amp-2**) (Scheme 3), and the concurrent H_2_ production (16.865 mol _H2_/mol_amp‑DNIC_ per 24 h; 75.3 H_2_-mmol/g_amp‑DNIC_ per 24 h) accompanied by the gradual reduction of N_2_O to N_2_ (Figure 8).

**Figure 8:**
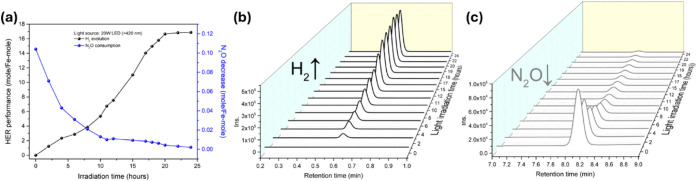
Time-dependent monitoring of H_2_ production and N_2_O consumption during the photodriven conversion of **amp-1**/Eosin Y/TEA to **amp-2**. (a) Time-dependent efficiency plot derived from GC data for (b) H_2_ generation and (c) N_2_O consumption.

**Scheme 3:**
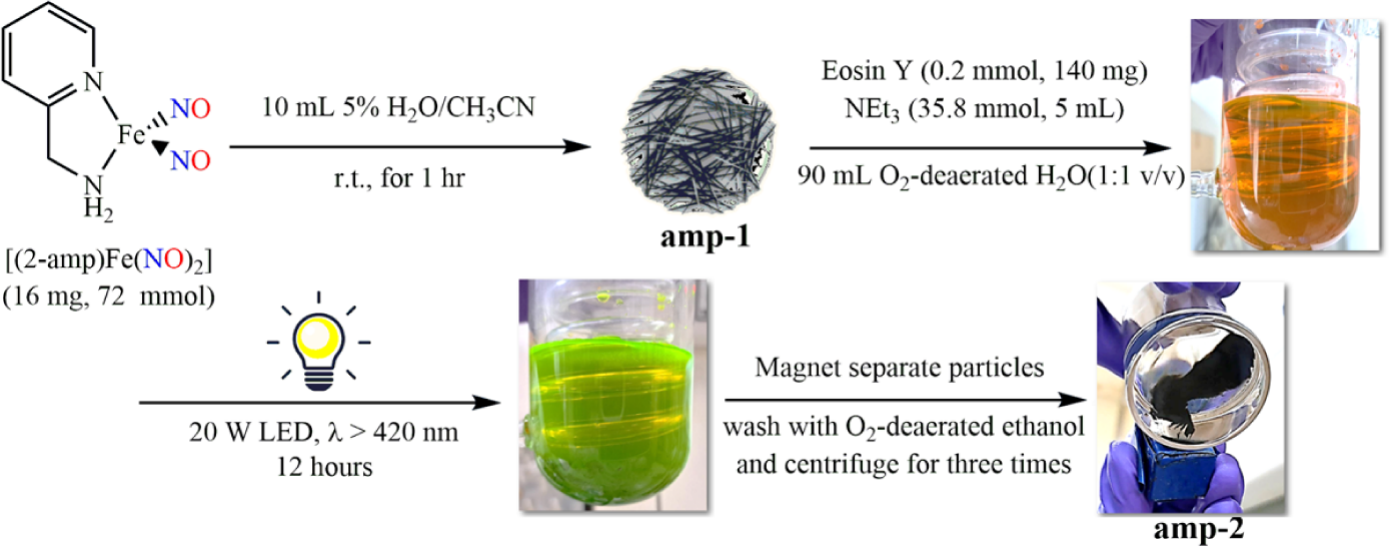
Photoinduced Reductive Conversion of **amp-1** into Fe@Fe_*x*_O_*y*_ Carbonaceous Particle (**amp-2**) along with H_2_ Evolution in the Presence of Eosin Y and Triethylamine

The suspended black **amp-2** particles were isolated as approximately 15 mg, and ICP-MS analysis of **amp-2** was conducted to determine the iron content to be 56.4%. FESEM images of **amp-2** exhibit nanoscale regular cubic particles with sizes ranging from 68 to 81 nm, as shown in Figure 4c,d. Compared to **amp-1**, the shrinkage/downsizing of particle size and shape change of **amp-2** may be attributed to the carboxylate-bearing Eosin Y and 2-A ligand acting as a “siderophore”, facilitating the (re)­dissolution of **amp-1** and subsequent photoinduced reduction of iron, yielding the regular cubic **amp-2**.^73,74^ EDX elemental analysis mapping of **amp-2** suggests that **amp-2** is primarily composed of C (32.41%), O (28.25%), and Fe (36.68%) with smaller amounts of N (2.21%), as displayed in Figure S16. The solid-state FTIR (Figure S12) and EDX mapping suggest that the degradation of Eosin Y into a fluorescent and other organic byproducts are deposited on the surface of **amp-2** particles after irradiation under anaerobic conditions.^75–77^ In contrast to the amorphous **amp-1**, the HRXRD pattern of **amp-2** emerges an apparent *Im*3̅*m* Fe^0^ pattern (at 17.59°, 24.98°, 30.71°, 35.62°, 39.96°, 43.99°, 47.72°, 54.61°, and 57.83 ^o^) along with an unapparent main peak of *Fd*3̅*m* Fe_3_O_4_ at 14.01°, as shown in Figure S17. The XPS DP atomic ratio of **amp-2** displays the increasing Fe component (from 5.8% to 40.3%) and the decreasing components of C (from 54.3% to 39.4%) and O (from 38.8% to 18.3%) with increasing etching depth (Figure 9a). Fe 2p XPS DP of **amp-2** reveals the significantly increasing component of metallic iron (binding energy at ∼706 eV) from the surface layer to the inner layer (Figure 9b). The XPS final inner layer fitting demonstrates that the compositions of Fe^0^, Fe^2+^, and Fe^3+^ are 52.2%, 30.9%, and 17.0%, respectively (Figure 9c). Fe K-edge XANES LCF analysis of **amp-2** implicates that the component of metallic iron is about 47.8 ± 1.4% (Figures 7a and 9d). The Fe K-edge EXAFS oscillation functions *k* ^3^χ­(*k*) of **amp-2**, ranging from 0 to 12 Å^–1^, exhibit a reduced oscillation amplitude, indicating that **amp-2** has better structural uniformity compared to **amp-1** (Figure 7c). The FT-EXAFS curve of **amp-2** displaying a conspicuous peak at 1.53 and 2.62 Å is assigned to the Fe–O bond and Fe···Fe distance, while the peak at 2.01 Å is assigned to the Fe–Fe bond of metallic iron (Figure 7c,d). Mössbauer spectra of **amp-2** show the isomer shifts (δ) at 0.12 mm/s (sextet) and 0.47 mm/s (doublet), respectively, assigned as metallic iron and Fe_*x*_O_*y*_ (Figure S14 and Table S1). In summary, the photoinduced transformation of **amp-1** to **amp-2** is due to the **amp-1** precursor continuously reducing Fe^II/III^ ions to form a crystalline zero-valence core.^74^ Simultaneously, water passivates Fe^0^ to create an amorphous iron oxide shell, resulting in a core–shell Fe@Fe_*x*_O_*y*_ (**amp-2**) along with hydrogen evolution.^78^ This transformation is characterized by a carbonaceous-coating core–shell cubic Fe@Fe_*x*_O_*y*_ (NZVI@NC),^72^ as evidenced by morphology, solid-state IR, Fe 2p XPS, XANES, and FT-EXAFS analysis.

**Figure 9:**
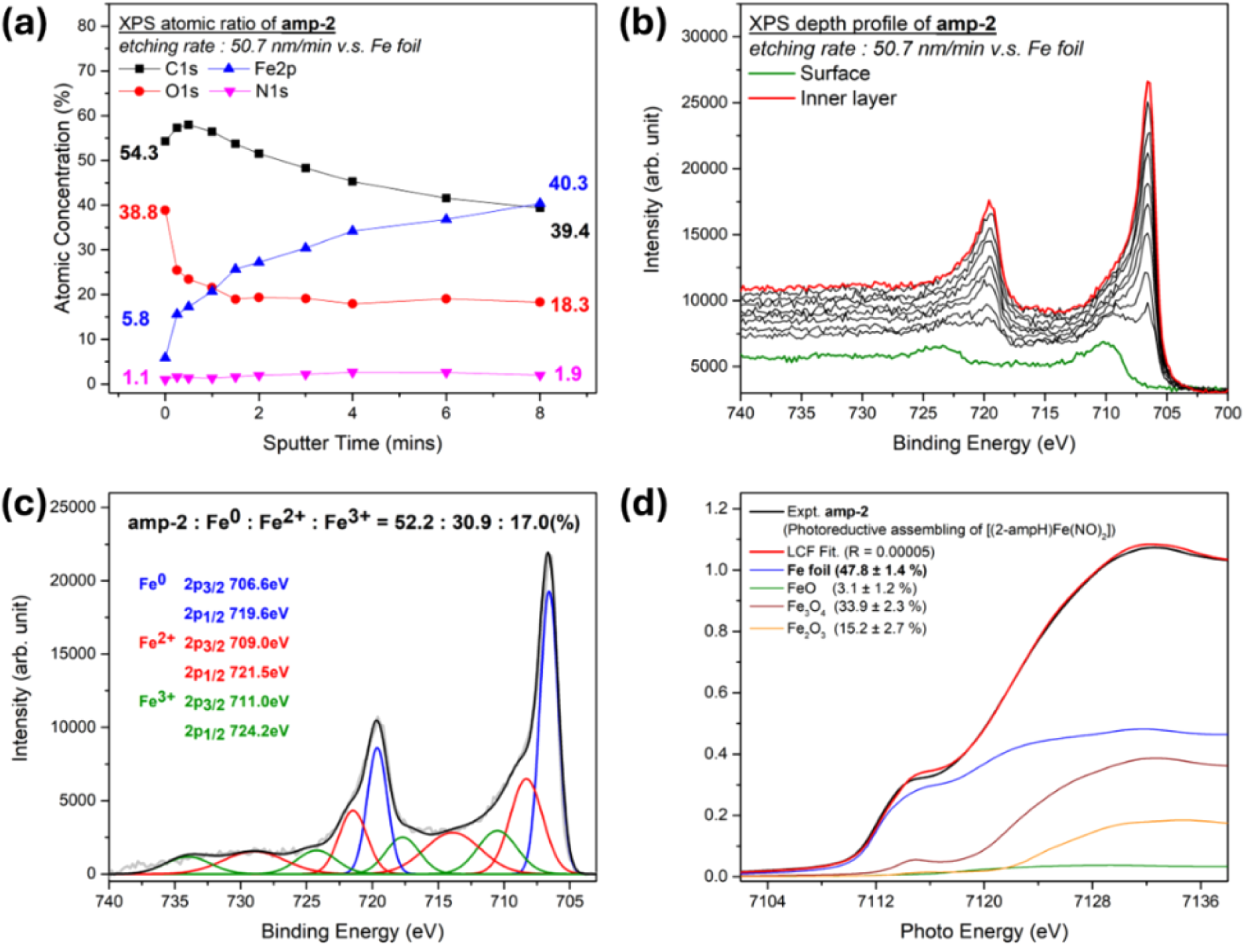
(a) XPS depth profile of atomic ratio for **amp-2** (etch rate: 50.7 nm/min vs Fe foil). (b) XPS Fe 2p spectra of **amp-2** at different etch depths. (c) XPS Fe 2p fitting for the inner layer of **amp-2**. (d) Fe K edge preedge XAS spectra of **amp-2** (black line) and its linear combination fitting for **amp-2**.

The photocatalytic hydrogen production system consists of recycled and redispersed NZVI@NC **amp-2**, Fl (0.2 mM), and 5% TEA (pH 11.0) in an aqueous solution (100 mL). The photocatalytic experiments for the subsequent cycles were conducted in a 200 mL photoreactor equipped with a Graham condenser, irradiated under λ > 420 nm LED (20 W) for 24 h per cycle. As shown in Figure S18, the photocatalytic hydrogen evolution over three cycles shows a decline in activity. In the first reused cycle (black curve), the catalyst exhibiting the highest efficiency with 72.3 mmol of H_2_/g over a 24-h period indicates the best activity. The second cycle (red curve) with hydrogen production of 58.6 mmol-H_2_/g per 24 h and the third cycle (blue curve) with 36.1 mmol-H_2_/g per 24 h display the gradual deactivation of the photocatalyst over time. This declining efficiency and longevity can be attributed to the gradual degradation of Eosin Y/TEA during photocatalysis, leading to the continuous adsorption of organic byproducts onto the catalyst surface, which blocks active sites and results in catalyst deactivation.^75^

## 3 Conclusion

This study uncovers that [NO]^−^-NO· release of nitrogen-bound mononuclear {Fe­(NO)_2_}^10^ [(2-A)­Fe­(NO)_2_] (**amp-DNIC**) was triggered by either receptor-mediated or receptor-free hydrolytic transformation pathways. Upon the addition of [Mn­(TPP)­(Cl)] into the THF solution of **amp-DNIC** in a 1:1 molar ratio, [Mn­(TPP)­(Cl)] mediates the transportation of [NO]^−^ and the subsequent ·NO from **amp-DNIC** to yield [Mn­(TPP)­(NO)] and then [Mn­(TPP)­(NO)_2_]. In contrast, the receptor-free acetonitrile solution of **amp-DNIC** containing 5% deaerated water results in the hydrolytic transformation of **amp-DNIC** to Fe_*x*_O_*y*_-type **amp-1** particles, along with the release of ·NO, N_2_O, and nitrite, as characterized by GC, IR, UV–vis, ICP-MS, XPS, FESEM, and EDX. Presumably, the transformation of **amp-DNIC** to **amp-1** proceeds via a hydroxide-bound intermediate [(2-A-H)­Fe­(OH)­(NO)_2_], enhancing the polarization of the Fe–NO bond in the DNIU to destabilize its Fe–NO bond and trigger the generation of ·NO, [NO]^−^, and Fe_*x*_O_*y*_. This study demonstrates that the complex [Fe­(NO)_2_]^10^ **amp-DNIC** acts as a bifunctional ·NO and [NO]^−^ donor, either in the THF solution of the [Mn­(TPP)­(Cl)] mediator or in the ACN-H_2_O solution, comparable to {Fe­(NO)_2_}^9^–{Fe­(NO)_2_}^9^ [Fe­(NO)_2_(SR)]_2_ RREs. In addition, the photoreduction of **amp-1** in the presence of Eosin Y and TEA leads to cubic Fe@Fe_*x*_O_*y*_ **amp-2** nanoparticles (Fe^0^ ∼ 48%) accompanied by hydrogen evolution. The process involves the hydrolysis of nitrogen-bound [Fe­(NO)_2_]^10^ DNIC, forming **amp-1** accompanied by the release of ·NO and NO^–^. The subsequent redissolution of **amp-1** particles and concurrent reductive reconstruction generate **amp-2** nanoparticles along with H_2_ production (Scheme 1c–f).

## 4 Experimental Section

All manipulations, reactions, and transfers were conducted under a pure nitrogen atmosphere according to Schlenk techniques or in a glovebox (N_2_ atmosphere). For inorganic compound synthesis, of solvents were purified and distilled under nitrogen by utilizing suitable reagents (diethyl ether/*n*-hexane/tetrahydrofuran (THF) from sodium benzophenone and acetonitrile from CaH_2_/P_2_O_5_/CaH_2_) and stored in dried, N_2_-filled flasks over 4 Å molecular sieves. 2-Aminomethylpyridine (2-A), Fe­(CO)_2_(NO)_2_, and [(2-A)­Fe­(NO)_2_] were synthesized by literature procedures.^37,79^ The reagents Mn­(TPP)Cl (Sigma-Aldrich), Co­(TPP) (Sigma-Aldrich), TBAOH (Sigma-Aldrich), Eosin Y (Chem-Impex Int.), and triethylamine (Sigma-Aldrich) were used as received. Infrared spectra (IR) were recorded on a PerkinElmer-Frontier instrument with sealed solution cells (0.1 mm, KBr window). UV–vis spectra were recorded with an Agilent 8453 spectrometer in a septum-sealed 1 cm path gas-tight quartz cuvette, and all reactions were carried out under a nitrogen atmosphere. ^1^H NMR spectra were obtained on a Varian Unity-500 MHz spectrometer. Analyses of carbon, hydrogen, and nitrogen were performed with a CHN analyzer (Elementar Vario EL III CHN-OS Rapid). Gaseous products were detected by Shimadzu GC-2030 gas chromatography, equipped with a barrier discharge ionization detector (BID). Powder X-ray diffraction (PXRD), morphologies (FESEM-EDX), and X-ray photoelectron spectroscopy (XPS) of iron-based particles (**amp-1** and **amp-2**) were studied as described below. The PXRD pattern collections were performed using TPS 19A (high-resolution X-ray diffraction; the energy was set to 20 keV) and a Bruker D2 Phaser (Cu Kα, λ = 0.154060 nm). The morphologies of the resulting iron-based particles were loaded onto a Si substrate and imaged using a field-emission scanning electron microscope (FESEM, JEOL JSM-7000F equipped with an Oxford EDX system), respectively. XPS measurements of the resulting iron-based particles were loaded on a Si substrate and analyzed on a ULVAC-PHI XPS spectrometer with a monochromatic Al anode as the X-ray source. The XPS depth profile of iron-based particles was carried out by 5 kV argon bombardment with a sputter rate of 50.7 nm/min for Fe. XPS data analysis and peak deconvolution using Gaussian–Lorentzian curve fitting based on Shirley background correction were accomplished with OriginPro and CASA XPS software.^80^

### 4.1 Safety Considerations

Dinitrosyl iron complexes have the potential to release toxic and/or odorless gases such as nitric oxide (NO) and nitrous oxide (N_2_O). Therefore, all reactions and storage involving these complexes must be conducted within a well-functioning fume hood and a nitrogen (N_2_)-filled glovebox. It is recommended to carry out these experiments during regular working hours to ensure that, in the event of exposure, an appropriate emergency response can be promptly initiated. Before any vessels are pressurized, including the glovebox, Schlenk line, and other containers, it is crucial to thoroughly inspect them for any cracks or damage. Additionally, these vessels should be properly secured using clamps, weights, or other appropriate methods to prevent potential accidents or incidents.

### 4.2 Preparation of [(2-A)­Fe­(NO)_2_] DNIC

The {Fe­(NO)_2_}^10^ [(2-A)­Fe­(NO)_2_] DNIC (**amp-DNIC**) was synthesized by literature procedures.^37^ A THF solution of [Fe­(CO)_2_(NO)_2_] prepared from the reaction of [K-18-crown-6-ether]­[Fe­(CO)_3_(NO)] (1.46 g, 3.0 mmol) and [NO]­[BF_4_] (0.348 g, 1.0 mmol) was transferred to a flask loaded with 2-(aminomethyl)­pyridine (0.36 mL, 3.6 mmol), and the reaction solution was stirred for 1 h under N_2(g)_ at ambient temperature. The resulting solution, gradually converted from brown to deep green, was then monitored by FTIR. The appearance of IR ν_NO_ stretching frequencies at 1694 and 1642 cm^–1^ was suggested as a characteristic feature of **amp-DNIC**. Then, *n*-hexane (20 mL) was added to the resulting solution to precipitate the dark green powder (isolated yield: 450 mg, 61%).

### 4.3 Reaction of **amp-DNIC** and [Co­(TPP)]

The THF solution of **amp-DNIC** (10 mM) and various equivalents of THF solution of [Co­(TPP)] (10–20 mM) were mixed at ambient temperature. Monitoring the appearance and fluctuation of IR ν_NO_ features (1694, 1642 cm^–1^) of **amp-DNIC** are plotted in Figure S2.

### 4.4 Addition of [Mn­(TPP)­(Cl)] into **amp-DNIC** Solution Leading to Nitric Oxide–Nitroxyl Release

The THF solution of [Mn^III^(TPP)­(Cl)] (3.6 mL, 0.05 mM) and the THF solution of **amp-DNIC** (0.4 mL, 0.05 mM) were mixed at ambient temperature. UV–vis spectra of absorbance at 587 and 623 nm, 570 and 605 nm, and 540 and 575 nm identify the generation of [Mn^III^(TPP)­(Cl)], [Mn­(TPP)­(NO)], and [Mn^III^(TPP)­(NO)_2_], respectively. IR ν_NO_ stretching frequencies at 1750 and 1742 cm^–1^ also suggest the formation of [Mn^III^(TPP)­(NO)_2_] and [Mn­(TPP)­(NO)], respectively. All reactions were followed until no more spectroscopic changes were observed, unless stated otherwise. Reaction time intervals ranged from 0 s to 2 h. The initial spectrum and each change were plotted in Figure 1.

The attempt to detect HNO release through the reductive ligation of triphenylphosphine was unsuccessful. An acetonitrile solution of triphenylphosphine (19 mg, 0.072 mmol) was added to the freshly dissolved **amp-DNIC** (16 mg, 0.072 mmol) in 2 mL of 5% water in d^4^-acetonitrile for 12 h. Reaction of PPh_3_ with **amp-DNIC** resulted in the formation of the water-stable complex [(PPh_3_)­Fe­(NO)_2_] DNIC. Then, the aliquot (1 mL) was analyzed by ^30^P NMR (δ = 60.8 ppm for [(PPh_3_)_2_Fe­(NO)_2_] and 26.9 ppm for PPh_3_=O were observed, but not δ = 30.8 ppm for PPh_3_=^14^NH).

### 4.5 Hydrolysis of **amp-DNIC** Forming Fe_*x*_O_*y*_ Particle (**amp-1**)

The **amp-DNIC** (16 mg, 72 μmol) was transferred into a 100 mL vial capped with a gastight rubber stopper under a N_2_ atmosphere. Deaerated 5% water in acetonitrile (10 mL) was added to the reaction vial, and then, the solution was vigorously stirred for 1 h, leading to a brownish colloidal suspension. The decreasing intensity of stretching frequencies (1694, 1642 cm^–1^) accompanied by the formation of N_2_O (2228 cm^–1^), monitored by FTIR (acetonitrile with 5% H_2_O), was observed, as shown in Figure 3a. The suspension solution was centrifuged with methanol three times and dried under vacuum. The isolated iron-based particles **amp-1** (12 mg) were stored in a N_2_-filled glovebox for characterization. The content of residual iron in the supernatant and the content of iron in **amp-1** were characterized as 1.11 ppm (26%) and 2.12 ppm (46%) via ICP-MS, respectively.

### 4.6 Reaction of **amp-DNIC** with TBAOH in Acetonitrile and THF Solution

**amp-DNIC** (22.4 mg, 0.1 mmol) and TBAOH (80 mg, 0.1 mmol) were loaded into a 20 mL vial individually, and 5 mL of deaerated and anhydrous selected solvent (acetonitrile and THF) was added. The vial was capped with a gastight rubber stopper under a N_2_ atmosphere. Each 1.0 mL of the prepared solutions of **amp-DNIC** and TBAOH was separately transferred into the same vial and rapidly mixed using a stir bar for FTIR measurements (Figures S7 and S10).

### 4.7 UV–Vis Spectroscopic Monitoring of **amp-DNIC** and TBAOH Reaction in Acetonitrile at −40 °C

Time-trace low-temperature UV–vis spectra measurements were performed using an Agilent 8453 spectrophotometer equipped with a UNICOKU liquid N_2_ cryostat. A freshly prepared solution (acetonitrile or THF) of **amp-DNIC** (1.0 mM, 4 mL) was transferred to a gastight quartz cell with a mini stirring bar and then cooled to −40 °C prior to the addition of 1 equiv of TBAOH (20 mM, 0.2 mL). Reaction time intervals ranged from 0 s to 5 h. The initial spectrum and change are plotted in Figure S8.

### 4.8 Determination of the Liberated NO/N_2_O Gases by GC-Bid

Quantification of NO and N_2_O were performed on a Shimadzu GC-2030 gas chromatograph equipped with a barrier discharge ionization detector (BID) and ShinCarbon ST (100/120 mesh) columns filled with poly­(dimethyl)­siloxane (stationary phase, Rtx-1). Helium was adopted as the mobile phase to conduct sample separation. The oven temperature was maintained at 40 °C and then raised up to 240 °C (a rate of 20 °C/min, and the detector was heated to 280 °C. The calibration curves were derived by injecting various amounts of pure N_2_O (0.10, 0.15, 0.20, 0.30, and 0.50 mL) into a vial (65 mL) containing 10 mL of ACN solution containing 5% H_2_O (v/v), followed by standing for 1 h, respectively. The calibration curve was plotted, as shown in Figure S3. Identification of the released NO and N_2_O are based on the gas chromatogram with retention times of 2.3 and 8.2 min, respectively (Figure 3c).

### 4.9 Tube-To-Cell NO Gas Detection via Trapping Agent [Co­(TPP)]

A two-armed reaction vessel-cuvette equipped with a Teflon roto-flow valve was loaded with **amp-DNIC** on the tube side and [Co­(TPP)] on the cell side under a nitrogen atmosphere (Figure S4).^61^ The detection of NO was conducted by adding deaerated water to the tube side containing **amp-DNIC** (0.0125, 0.025, and 0.030 mM). The yield of NO was estimated by its diffusion from the tube side into a quartz cell containing 0.05 mM [Co­(TPP)] in THF. The reaction transformation was monitored by the increase of absorbance at 540 nm ([Co­(NO)­(TPP)]) and the decrease at 520 nm ([Co­(TPP)]), as shown in the UV–vis spectroscopy plots in Figures S3 and S4. Time-trace UV–vis spectra are plotted in Figure S4, and the reaction reaches completion after 120 min. During the hydrolysis process, NO gas diffuses within the sealed two-armed reaction vessel-cuvette, ensuring that no side reactions occur on the cuvette side. As a result, only [Co­(TPP)] on the cuvette side reacts with the released NO. Thus, using [Co­(TPP)] and [Co­(TPP)­(NO)] as standard references under the known concentration (0.05 mM), the yield of NO release can be determined through linear combination fitting of the postreaction UV–vis spectra. Data analysis was based on the linear combination of the ratio of the [Co­(TPP)]/[Co­(NO)­(TPP)] spectrum. The yield of NO from the hydrolysis of **amp-DNIC** was estimated as 75.8 ± 3.7%.

### 4.10 Griess Test of the Liberated Nitrite from Hydrolysis of [(2-A)­Fe­(NO)_2_] in Water/ACN (0.5:9.5 v/v)

Quantification of the released nitrite was completed via the Griess reagent test (the feature peak (absorbance at 540 nm) recorded by an Agilent 8453 UV/vis spectrophotometer). The Griess test solution was prepared by weighing 35 mg of modified Griess reagent powder (Sigma G4410) and then dissolved with 50 mL of deaerated water. The calibration curves were derived using various concentrations of sodium nitrite solution (0, 5.6, 7.5, 15, 22.5, and 30 μM) and were plotted as shown in Figure S6. The produced nitrite was isolated via centrifugation, and the supernatant was quantified by the Griess test (yield: 6.1 ± 0.4%, 2.8 μmol).

### 4.11 Reductive Assembly of Fe@Fe_*x*_O_*y*_ Carbonaceous Particle (**amp-2**) and HER via Photolysis of Xanthene Dye, [(2-A)­Fe­(NO)_2_], and Triethylamine

The 90 mL of deaerated water solution of Eosin Y (0.2 mmol, 140 mg) and triethylamine (35.8 mmol, 5 mL) was added to the 200 mL photoreactor containing the freshly prepared 10 mL of 5% water-in-acetonitrile (1:1 v/v) solution of [(2-A)­Fe­(NO)_2_] DNIC (16 mg, 0.072 mmol). Then, a 20 W LED light (λ > 400 nm) was adopted to irradiate the mixture solution with vigorous stirring at 25 °C for 24 h. The resulting magnetic particles were washed/centrifuged with methanol three times and then dried under vacuum. The isolated magnetic particles **amp-2** (15 mg) were stored in a N_2_-filled glovebox for characterization. ICP-MS analysis showed 0.86 ppm (12%) residual iron in the supernatant and 4.32 ppm (56.4%) iron in **amp-2**. The headspace gas of the in situ produced H_2_ (0.82 min) per hour and hydrogen production vs. N_2_O consumption under photoreduction of Eosin Y, TEA, and **amp-1** are plotted in Figure 9.

### 4.12 Recycling Fe@Fe_*x*_O_*y*_ Carbonaceous Particles (**amp-2**) for Photocatalytic HER

After each HER cycle (synthesis of **amp-2** and reuse of photocatalytic HER), the resulting particles were washed, sonicated, and centrifuged with deaerated methanol three times, dried under vacuum, and then isolated. The isolated dry particles (10 mg) and Eosin Y (0.2 mmol, 140 mg) were weighed in a 200 mL photoreactor, followed by the subsequent addition of 95 mL of deaerated water and triethylamine (35.8 mmol, 5 mL) under a N_2_ atmosphere. Then, a 20 W LED light (λ > 400 nm) was adopted to irradiate the mixture solution with vigorous stirring at 25 °C for 24 h. The headspace gas of the in situ produced H_2_ (0.82 min) per 4–8 h, and the calculated HER performances were plotted in Figure S17.

### 4.13 X-ray Absorption Spectroscopy (XAS)

X-ray absorption experiments were carried out at the National Synchrotron Radiation Research Center (NSRRC), Hsinchu, Taiwan, and Super Photon ring-8 (SPring-8), Hyogo, Japan. Samples were ground to powder and secured in a bag made of 6-μm Mylar film. The Fe K-edge X-ray absorption spectroscopy data were collected in fluorescence mode on BL17C and BL12B2 with a double-crystal Si(111) monochromator. The energy resolution Δ*E*/*E* was estimated to be about 2 × 10^–4^. High harmonics were rejected by Rh-coated mirrors. The energy is scanned from 6.912 to 7.609 keV. A Lytle detector was employed for the fluorescence measurements. The photon energy was calibrated to the maximum of the first inflection point at 7112.0 eV in the Fe foil spectrum. All data are calibrated, analyzed, and fitted using Athena software.^81^ Data were averaged, and a smooth background was removed from all spectra by fitting a straight line to the preedge region and subtracting this straight line from the entire spectrum. Normalization of the data was accomplished by fitting a flat polynomial to the postedge region and normalizing the edge jump to 1.0.^81^

### 4.14 Mössbauer Measurements

Using a SeeCo constant acceleration spectrometer equipped with a temperature controller maintaining temperatures within ± 0.1 K and a 57Co radiation source in a Rh matrix, zero-field^57^ Fe Mössbauer spectra of **amp-DNIC**, **amp-1**, and **amp-2** were recorded at 295 K. All measurements were performed under a N_2(g)_ atmosphere. Isomer shifts refer to α-Fe metal at room temperature. Data were fitted with a sum of Lorentzian quadrupole doublets by using a least-squares routine with the WMOSS program.^82^

## Supplementary Material



## Notes

Additional experimental details and spectra, including photographs of the experimental setup (PDF)
