# Supplementary material for: Receptor-Mediated and Hydrolytic Denitrosylation of Dinitrosyl Iron Complexes to Yield Amorphous Fe x O y and Its Photoinduced Transformation into Crystalline Fe@Fe x O y Nanoparticles
Source: Inorg Chem. 2025 Jun 17;64(25):12708–21. doi: 10.1021/acs.inorgchem.5c01434 (PMC12216227; doi:10.1021/acs.inorgchem.5c01434)
Supplement: Supplementary file 1 [file ic5c01434_si_001.pdf]

Supporting Information

Receptor-mediated and Hydrolytic Denitrosylation of Dinitrosyl Iron Complexes to Yield Amorphous Fe_xO_y and Its Photo-induced Transformation into Crystalline $Fe@Fe_xO_y$ Nanoparticles

Wun-Yan Wu,[a] Yu-Shen Lin,[a], Linda Iffland,[b,c] Ulf-Peter Apfel,[b,c] Tsai-Te Lu,[a,d]
Wen-Feng Liaw*[a]

[a] Department of Chemistry, National Tsing Hua University, Hsinchu 30013, Taiwan
[b] Department of Chemistry and Biochemistry, Activation of Small Molecules/Technical Electrochemistry, Ruhr-Universität Bochum, 44801 Bochum, Germany.
[c] Department of Electrosynthesis, Fraunhofer UMSICHT, 46047 Oberhausen, Germany
[d] Institute of Biomedical Engineering, National Tsing Hua University, Hsinchu 30013, Taiwan

E-mail address:
wfliaw@mx.nthu.edu.tw (WFL)

Table of Contents

Figure S1. Mössbauer spectrum of $\{Fe(NO)_2\}^{10}$ [(2-amp)$Fe(NO)_2$] DNIC. **S4**

Figure S2. FTIR spectra (THF) show the stretching frequencies of 10 mM **amp-DNIC** at 1694, 1642 cm^{-1} (black line), reaction of **amp-DNIC** and 1.0 equivalent of [Co(TPP)] (red line), 2.0 equivalent of [Co(TPP)] (blue line) and 10 mM [Co(TPP)] (light purple line). **S4**

Figure S3. Calibration curves for quantification of N_2O **S5**

Figure S4. (a) UV-vis spectra of time-dependent tube-to-cell •NO trapped by 0.05 mM [Co(TPP)] in THF. (NO release triggered by adding 5 % O_2 -deaerated H_2O into the acetonitrile solution of **amp-DNIC** (0.025 mM)). (b) Time-dependent produced NO diffused into [Co(TPP)] leading to [Co(TPP)(NO)], monitored by UV-Vis spectra (based on 540 nm) for 2 h. **S5**

Figure S5. (a-e) NO liberation yield estimated by NO diffusion from side tube (hydrolysis of various concentration of **amp-DNIC** (0.0125, 0.025 and 0.030 mM)) to quartz cell (0.05 mM [Co(TPP)] in THF). (f) Calculation of yield based on linear combination of the resulting spectrum. **S6**

Figure S6. (a-b) Calibration curve established by reactions of Griess reagent and the different concentrations of $NaNO_2$ in H_2O, and based on UV-Vis absorbance of 540 nm. (c) UV-vis spectra (entries 1-5) of hydrolysis of **amp-DNIC** in the acetonitrile solution containing 5 % H_2O (the suspended iron-based powder was removed via centrifugation). (d) Calculation of yield based on UV-Vis absorbance of 540 nm. ... **S6**

Figure S7. (a) FTIR spectra (acetonitrile) show the stretching frequencies of 10 mM **amp-DNIC** at 1689, 1635 cm^{-1} (black line), reaction of **amp-DNIC** and 1.0 equivalent of TBAOH after 0.5 min (red line), 1 min (blue line), 2 min (light purple line), 3 min (green line), 5 min (navy line) and 30 min (brown line). (b) FTIR spectrum (acetonitrile) show the stretching frequencies of 10 mM TBAOH for two hours at 1708, 1682 cm^{-1}. **S7**

Figure S8. Low temperature UV-Vis spectra (acetonitrile) of 1.0 mM **amp-DNIC** at –40 °C (black line), monitoring the reaction of **amp-DNIC** with 1.0 equiv. TBAOH at 30-second intervals (gray lines), and the reaction after 5 hours (red line). **S7**

Figure S9. (a) UV-Vis spectra (acetonitrile) of 1.0 mM **amp-DNIC** at ambient temperature (black line), the reaction of **amp-DNIC** with 1.0 equiv. TBAOH after 5 hours at –40 °C (red line), and the subsequent warming up to ambient temperature after

3 hours (blue line). (b) UV-Vis spectrum (acetonitrile) of 1.0 mM TBAOH at ambient temperature (black line). **S8**

Figure S10. FTIR spectra (THF) show the stretching frequencies of 10 mM **amp-DNIC** at 1694, 1642 cm^{-1} (black line), reaction of **amp-DNIC** and 1.0 equivalent of TBAOH after 3 min (red line), 5 min (blue line), 30 min (green line) and without **amp-DNIC** after 30 min (light pink line). **S8**

Figure S11. (a) SEM images and the corresponding EDX elemental mapping images of (b) C, (c) O, (d) N, (e) Fe and (f) component analysis of **amp-1**. **S9**

Figure S12. Solid-state FTIR spectra of **amp-1** (black), **amp-2** (red), 2-amp ligand (blue) and Eosin Y (green). The major peaks for 2-amp ligand (blue zoom) and Eosin Y (yellow zoom), respectively. **S9**

Figure S13. Fe 2p XPS spectra for the commercial Fe_3O_4 at different etch (etch rate: 50.7 nm/min versus Fe foil). **S10**

Figure S14. Mössbauer spectra (298 K) of (a) Fe_3O_4 standard, (b) Fe^0 standard, (c) **amp-1** and (d) **amp-2**. **Multiple trace amount peaks were not fitted into the spectra and only refer to the corresponding standards.* **S10**

Figure S15. (a) SEM images and the corresponding EDX elemental mapping images of (b) O, (c) Fe, (d) C, (e) N and (f) component analysis in **amp-2**. **S11**

Figure S16. Photo-durability test of **amp-DNIC** monitored by FTIR. Black line shows the acetonitrile solution of 8.0 mg **amp-DNIC**. Red line shows the acetonitrile solution of 8.0 mg **amp-DNIC** was irradiated by 20 W LED light ($\lambda > 430$ nm) for 24 h, leading to the partial decomposition. **S11**

Figure S17. Powder X-ray diffraction pattern of **amp-2** (black line), $Fd\overline{3}m$ Fe_3O_4 database (blue line) and $Im\overline{3}m$ zero valence iron database (red line). Experimental data collections were performed in high-resolution X-ray diffraction (energy set to 20 keV (λ = 0.61992 Å), TPS 19A, NSRRC, Taiwan). **S12**

Figure S18. Photocatalytic HER performance over three reuse cycles showing decreasing H_2 production efficiency from the first to the third cycle. **S12**

Table S1. Mössbauer parameters: isomer shift (δ), quadrupole splitting (ΔE_Q) and hyperfine field, obtained by fitting the spectra recorded at 298 K for standards, **amp-DNIC**, **amp-1** and **amp-2** **S13**

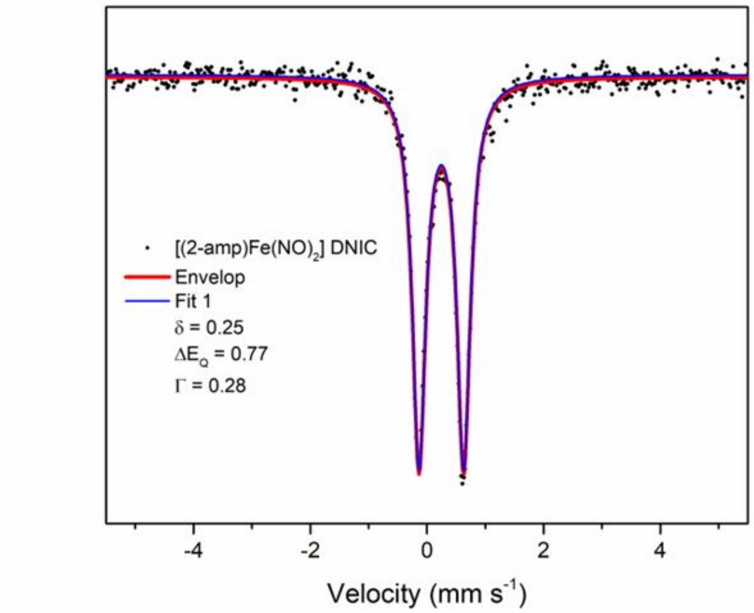

Figure S1. Mössbauer spectrum of $\{Fe(NO)_2\}^{10}$ [(2-amp)$Fe(NO)_2$] DNIC (black dot), component fit (blue line,) with $\delta = 0.25$ and $\Delta E_Q = 0.77$, and fit envelop (red line).

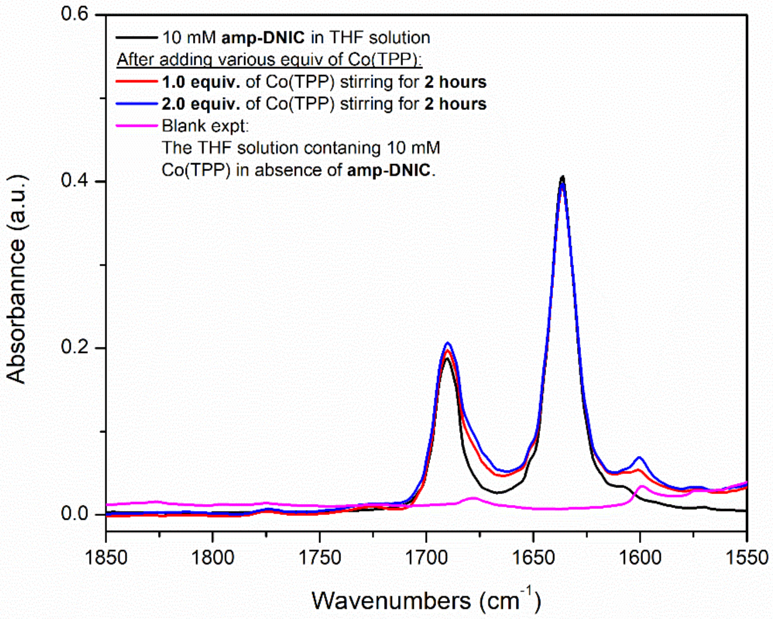

Figure S2. FTIR spectra (THF) show the stretching frequencies of 10 mM **amp-DNIC** at 1694, 1642 cm^{-1} (black line), reaction of **amp-DNIC** and 1.0 equivalent of [Co(TPP)] (red line), 2.0 equivalent of [Co(TPP)] (blue line) and 10 mM [Co(TPP)] (light purple line).

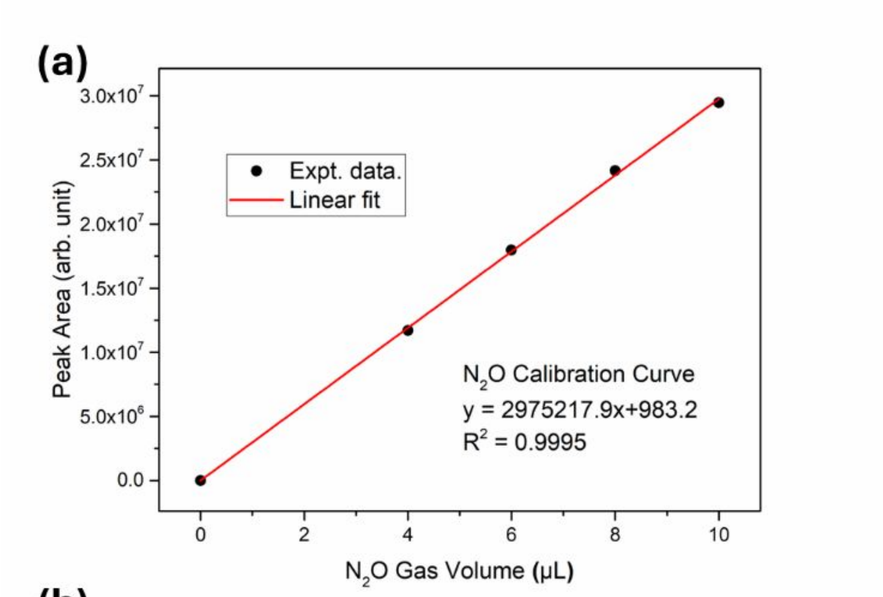

(b)

Entry	Area	mL	mmol	%
1	980541.4	0.329239	8.363	11.6
2	894300.2	0.300253	7.626	10.6
3	849432.8	0.285172	7.244	10.1
**Conversion of DNIU-to-N_2O: 10.8% +/- 0.8**				

Figure S3. (a) Calibration curves for quantification of N_2O established by injection of various amounts of N_2O (0.004, 0.006, 0.008 and 0.010 mL, individually). Detection of N_2O based on gas chromatogram (retention time at 8.14 min.). (b) Calculation of yield based on the GC plot area of the released N_2O.

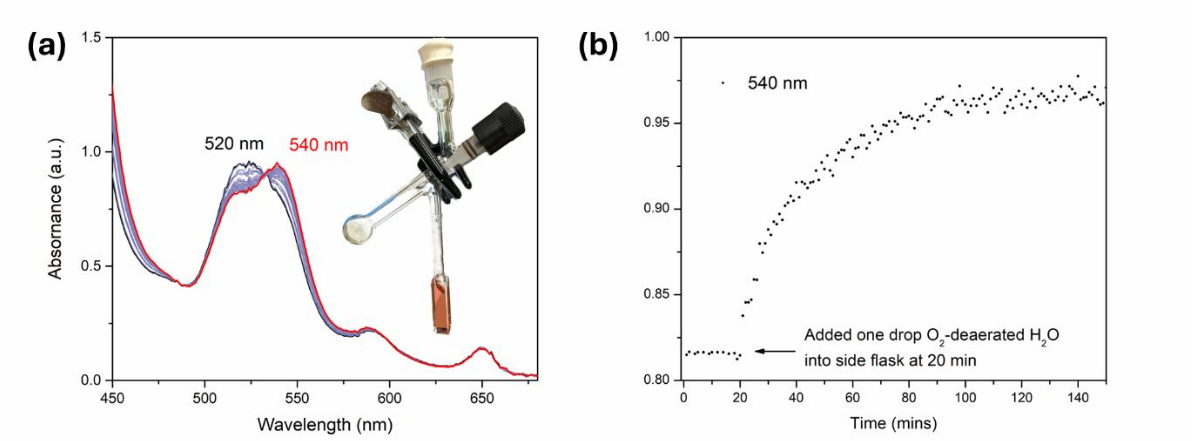

Figure S4. (a) UV-vis spectra of time-dependent tube-to-cell •NO trapped by 0.05 mM [Co(TPP)] in THF. (NO release triggered by adding 5 % O_2 -deaerated H_2O into the acetonitrile solution of **amp-DNIC** (0.025 mM)). (b) Time-dependent produced NO diffused into [Co(TPP)] leading to [Co(TPP)(NO)], monitored by UV-Vis spectra (based on 540 nm) for 2 h.

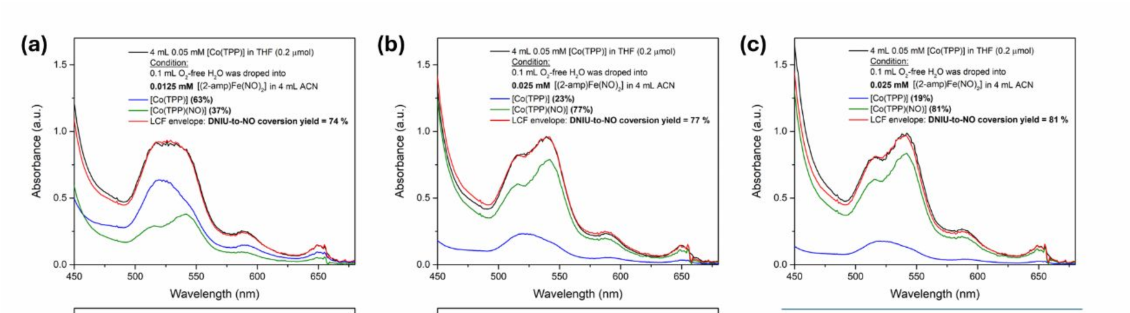

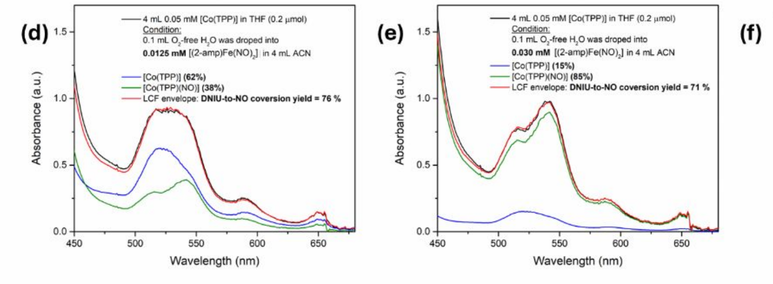

(f)

Entry	amp-DNIC (mM)	Co(TPP)/Co(TPP)(NO) (%)	NO release from amp-DNIC (%)
1	0.0125	63/37	74
2	0.0125	62/38	76
3	0.025	23/77	77
4	0.025	19/81	81
5	0.03	15/85	71
NO release yield: 75.8% +/- 3.7			

Figure S5. (a-e) NO liberation yield estimated by NO diffusion from side tube (hydrolysis of various concentration of **amp-DNIC** (0.0125, 0.025 and 0.030 mM)) to quartz cell (0.05 mM [Co(TPP)] in THF). (f) Calculation of yield based on linear combination of the resulting spectrum.

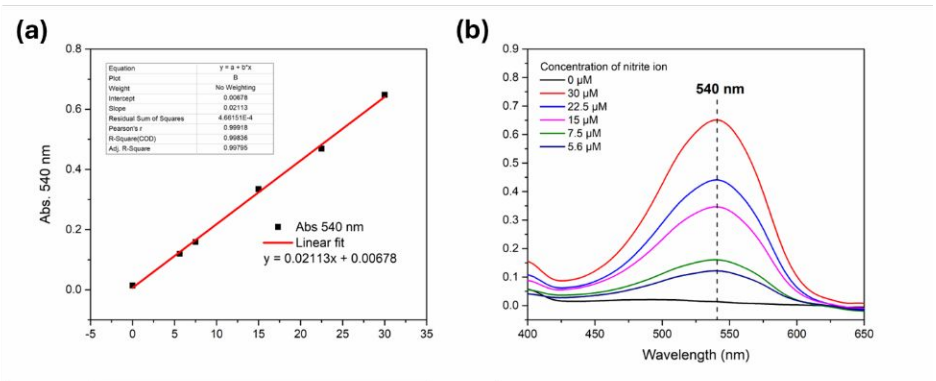

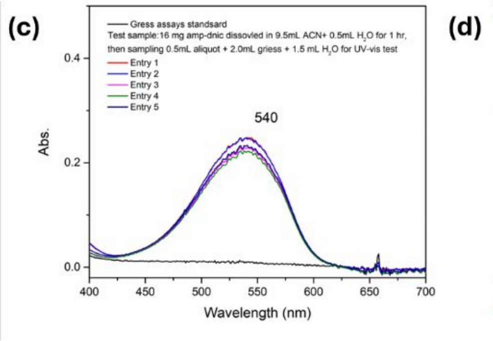

(d)

Entry	Amp-DNIC (mg)	Abs. 540nm	Nitrite conc. (mM)	Nitrite conversion (%)
1	16.0	0.247	0.0115	6.5
2	16.0	0.245	0.0114	6.4
3	16.0	0.227	0.0106	5.9
4	16.0	0.220	0.0103	5.7
5	16.0	0.231	0.0108	6.0
NO release yield: 6.1% +/- 0.4				

Figure S6. (a-b) Calibration curve established by reactions of Griess reagent and the different concentrations of $NaNO_2$ in H_2O, and based on UV-Vis absorbance of 540 nm. (c) UV-vis spectra (entries 1-5) of hydrolysis of **amp-DNIC** in the acetonitrile solution containing 5 % H_2O (the suspended iron-based powder was removed via centrifugation). (d) Calculation of yield based on UV-Vis absorbance of 540 nm.

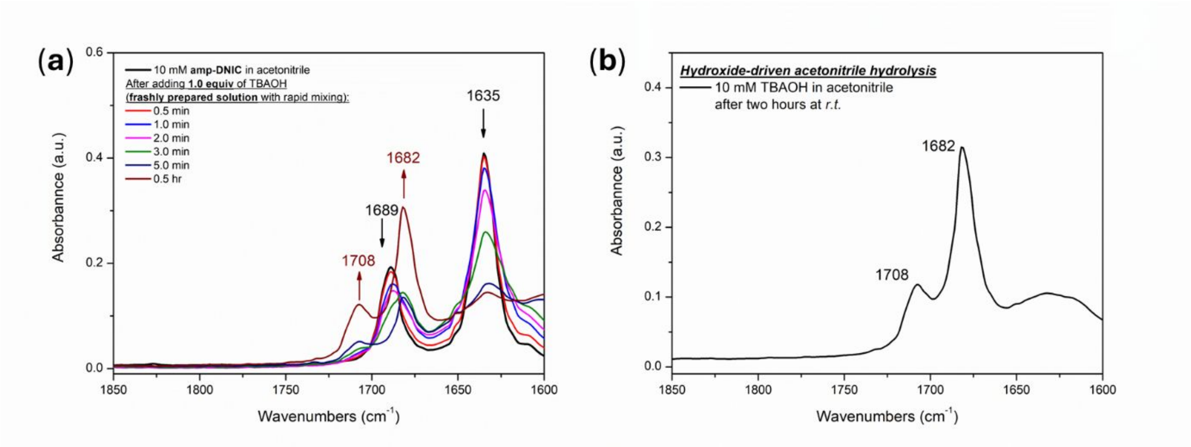

Figure S7. (a) FTIR spectra (acetonitrile) show the stretching frequencies of 10 mM **amp-DNIC** at 1689, 1635 cm^{-1} (black line), reaction of **amp-DNIC** and 1.0 equivalent of TBAOH after 0.5 min (red line), 1 min (blue line), 2 min (light purple line), 3 min (green line), 5 min (navy line) and 30 min (brown line). (b) FTIR spectrum (acetonitrile) show the stretching frequencies of 10 mM TBAOH for two hours at 1708, 1682 cm^{-1}.

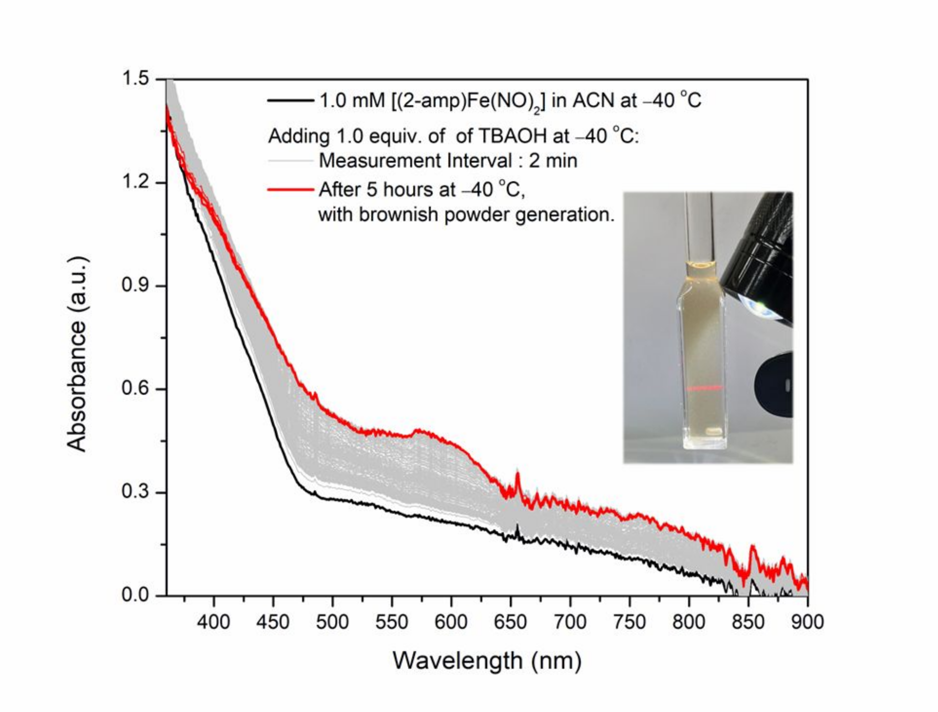

Figure S8. (a) Low temperature UV-Vis spectra (acetonitrile) of 1.0 mM **amp-DNIC** at –40 °C (black line), monitoring the reaction of **amp-DNIC** with 1.0 equiv. TBAOH at 30-second intervals (gray lines), and the reaction after 5 hours (red line). (b) UV-Vis spectra (acetonitrile) of 1.0 mM **amp-DNIC** at ambient temperature (black line), the reaction of **amp-DNIC** with 1.0 equiv. TBAOH after 5 hours at –40 °C (red line), and the subsequent warming up to ambient temperature after 3 hours (blue line).

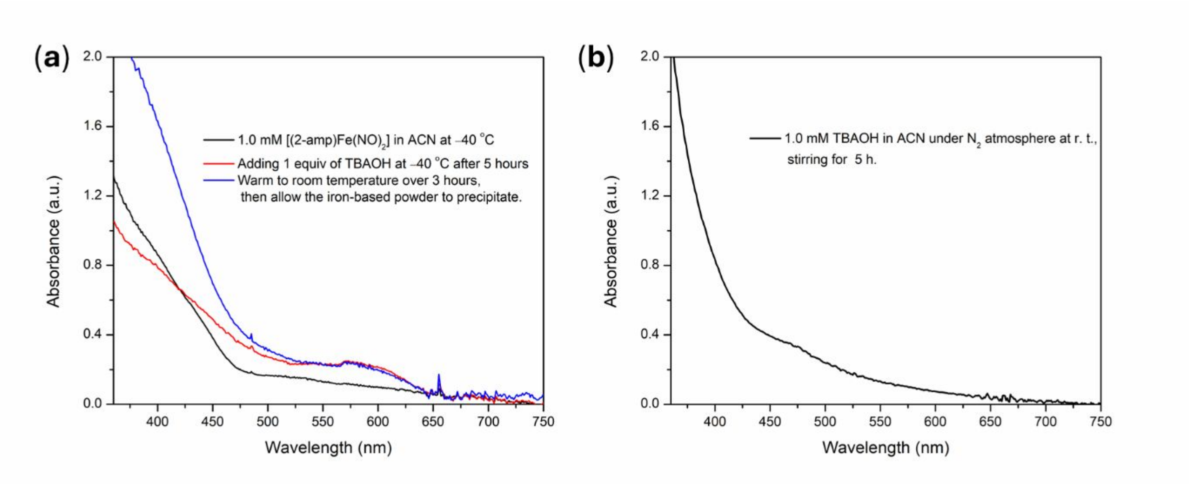

Figure S9. (a) UV-Vis spectra (acetonitrile) of 1.0 mM **amp-DNIC** at ambient temperature (black line), the reaction of **amp-DNIC** with 1.0 equiv. TBAOH after 5 hours at –40 °C (red line), and the subsequent warming up to ambient temperature after 3 hours (blue line). (b) UV-Vis spectrum (acetonitrile) of 1.0 mM TBAOH at ambient temperature (black line).

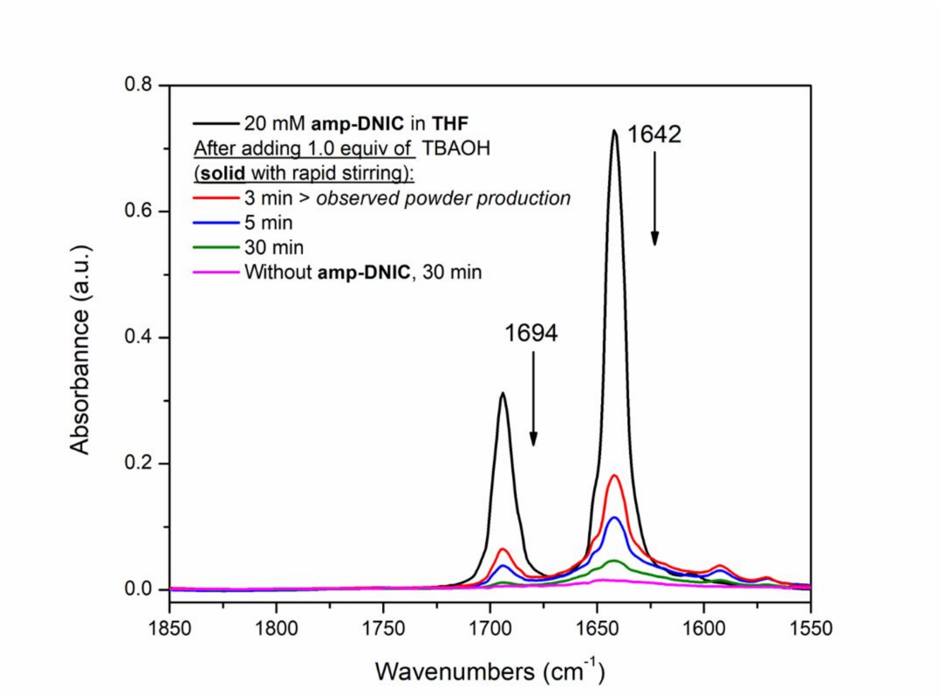

Figure S10. FTIR spectra (THF) show the stretching frequencies of 10 mM **amp-DNIC** at 1694, 1642 cm^{-1} (black line), reaction of **amp-DNIC** and 1.0 equivalent of TBAOH after 3 min (red line), 5 min (blue line), 30 min (green line) and without **amp-DNIC** after 30 min (light pink line).

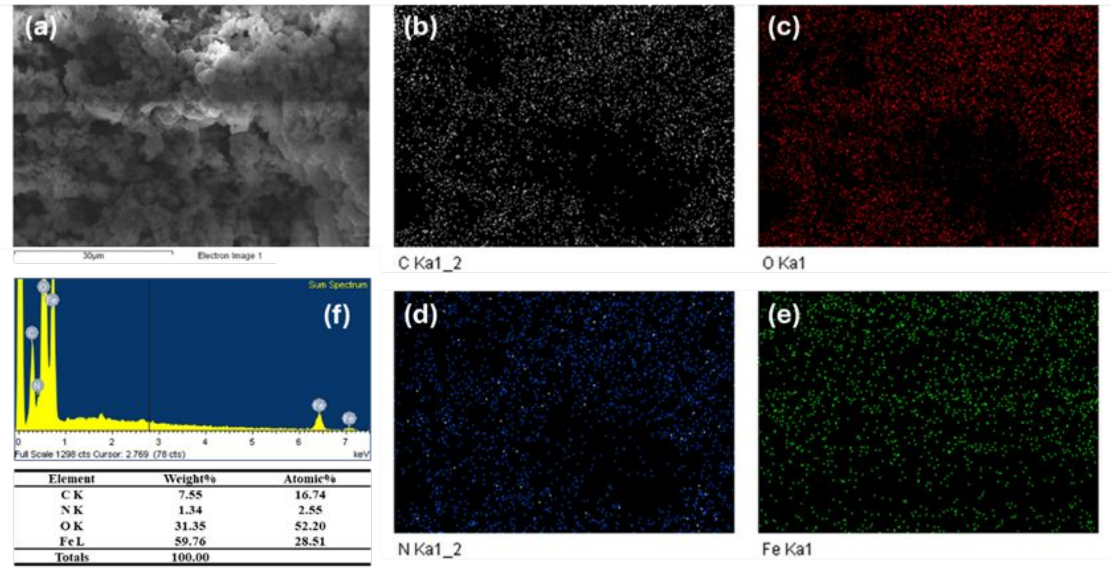

Element	Weight%	Atomic%
C K	7.55	16.74
N K	1.34	2.55
O K	31.35	52.20
Fe L	59.76	28.51
Totals	100.00	

Figure S11. (a) SEM images and the corresponding EDX elemental mapping images of (b) C, (c) O, (d) N, (e) Fe and (f) component analysis of **amp-1**.

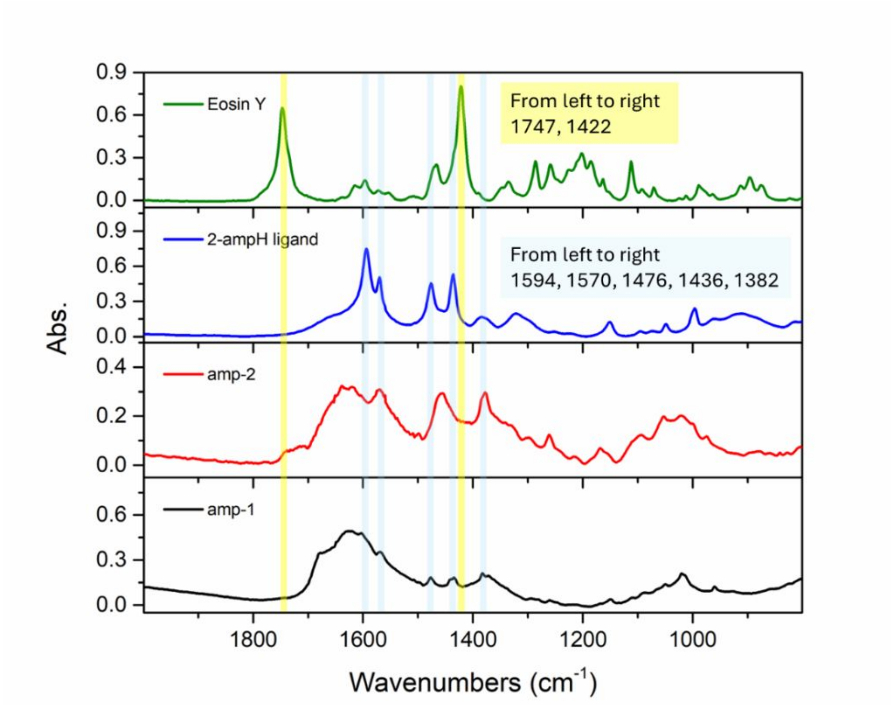

Figure S12. Solid-state FTIR spectra of **amp-1** (black), **amp-2** (red), 2-amp ligand (blue) and Eosin Y (green). The major peaks for 2-amp ligand (blue zoom) and Eosin Y (yellow zoom), respectively.

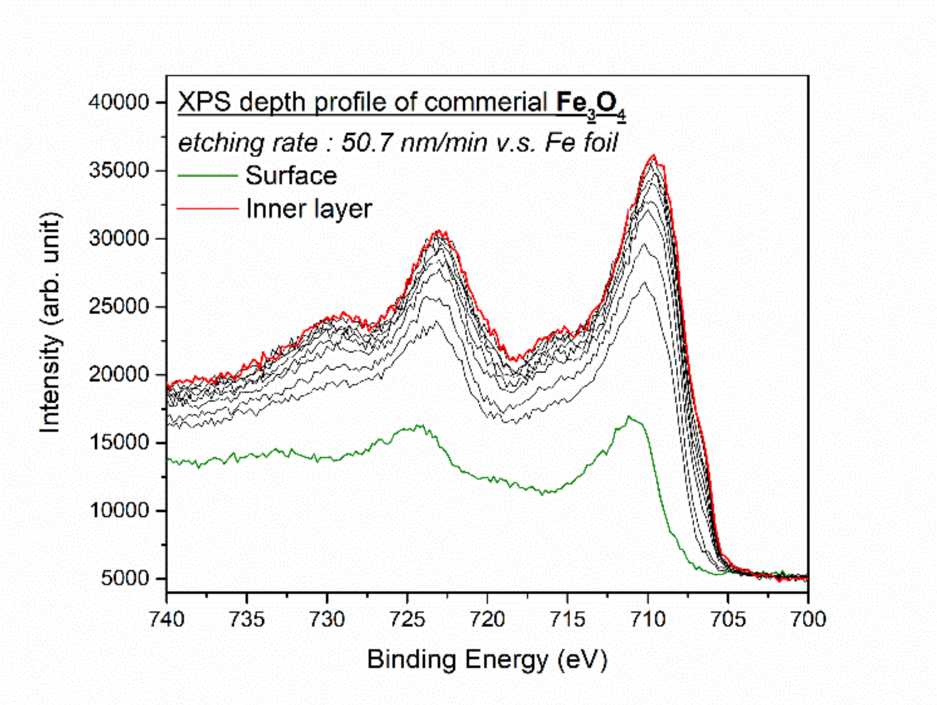

Figure S13. Fe 2p XPS spectra for the commercial Fe_3O_4 at different etch (etch rate: 50.7 nm/min versus Fe foil).

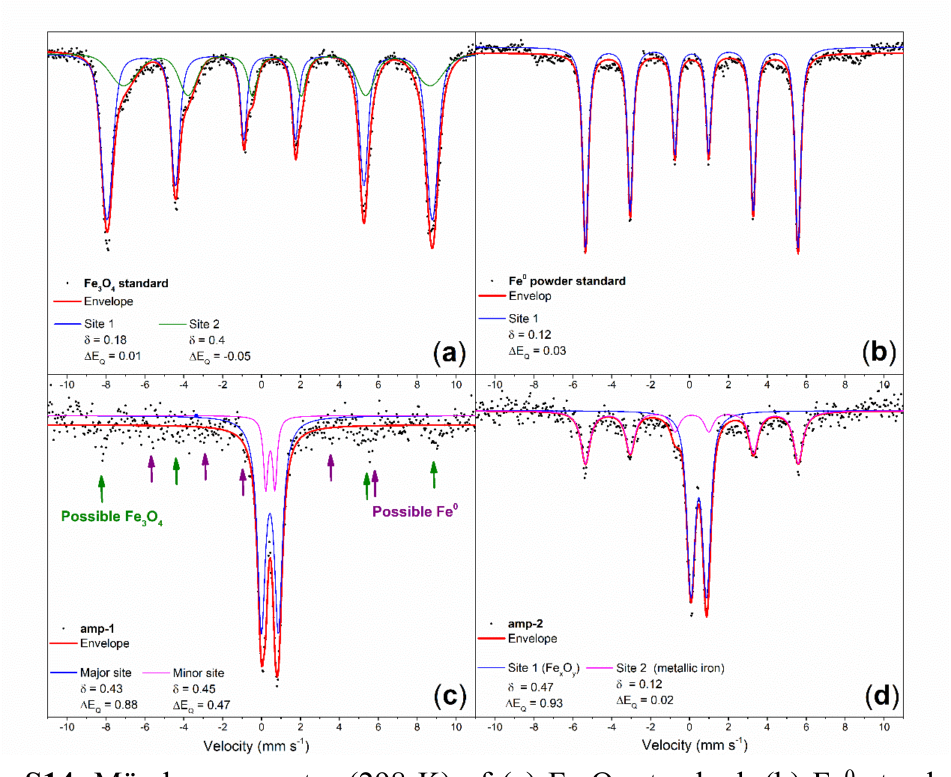

Figure S14. Mössbauer spectra (298 K) of (a) Fe_3O_4 standard, (b) Fe^0 standard, (c) **amp-1** and (d) **amp-2**. **Multiple trace amount peaks were not fitted into the spectra and only refer to the corresponding standards.*

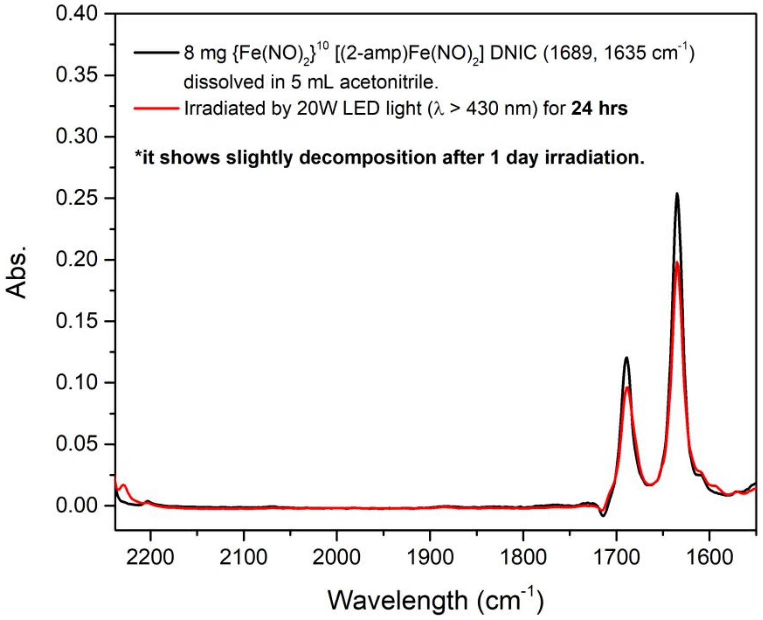

Figure S15. Photo-durability test of **amp-DNIC** monitored by FTIR. Black line shows the acetonitrile solution of 8.0 mg **amp-DNIC**. Red line shows the acetonitrile solution of 8.0 mg **amp-DNIC** was irradiated by 20 W LED light (λ > 430 nm) for 24 h, leading to the partial decomposition.

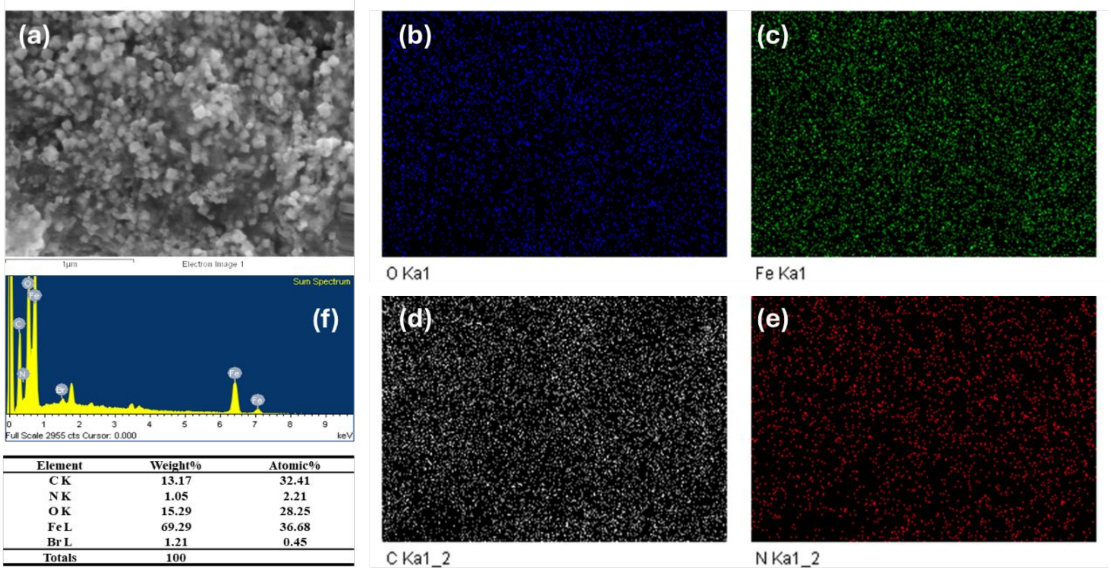

Element	Weight%	Atomic%
C K	13.17	32.41
N K	1.05	2.21
O K	15.29	28.25
Fe L	69.29	36.68
Br L	1.21	0.45
Totals	100	

Figure S16. (a) SEM images and the corresponding EDX elemental mapping images of (b) O, (c) Fe, (d) C, (e) N and (f) component analysis in **amp-2**.

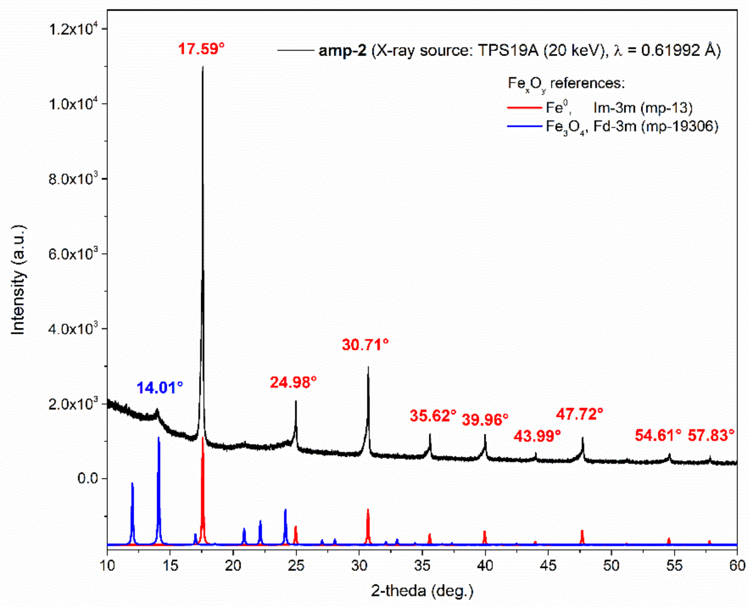

Figure S17. Powder X-ray diffraction pattern of **amp-2** (black line), $Fd\overline{3}m$ Fe_3O_4 database (blue line) and $Im\overline{3}m$ zero valence iron database (red line). Experimental data collections were performed in high-resolution X-ray diffraction (energy set to 20 keV (λ = 0.61992 Å), TPS 19A, NSRRC, Taiwan).

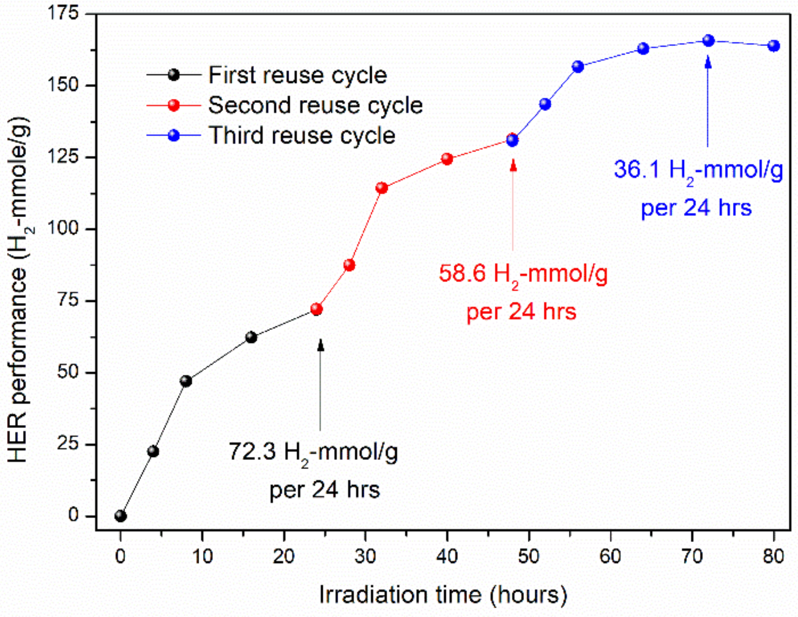

Figure S18. Photocatalytic HER performance over three reuse cycles showing decreasing H_2 production efficiency from the first to the third cycle.

Table S1. Mössbauer parameters: isomer shift (δ), quadrupole splitting (ΔE_Q) and hyperfine field, obtained by fitting the spectra recorded at 298 K for standards, **amp-DNIC**, **amp-1** and **amp-2**.

	**Isomer shift (δ, mm s^{-1})**	**Sextet (S) /Doublet (D)**	**Quadrupole splitting (ΔE_Q, mm s^{-1})**	**Assignment**
Standard Fe_3O_4	0.18	S	0.01	Magnetic-Fe^{2+}
	0.40	S	-0.05	Magnetic-Fe^{3+}
Standard Iron(0)	0.12	S	0.03	Fe^0
**amp-1**	0.43	D	0.88	Major: Fe_xO_y
	0.45	D	0.47	Minor: Fe_xO_y
**amp-2**	0.12	S	0.02	Fe^0
	0.47	D	0.93	Hs-Fe^{3+}
**$\{Fe(NO)_2\}^{10}$ [(2-amp)$Fe(NO)_2$]**	0.25	D	0.77	(*N, N*)-$\{Fe(NO)_2\}^{10}$
Reported Fe_xO_y species	0.34-0.48	D	0.32-0.83	Fe^{3+}/Fe^{2+}

Reference

(1) Powder X-ray diffraction database was downloaded from Material Project (https://next-gen.materialsproject.org/ ; Fe: mp-13; Fe_3O_4: mp-19306; Fe_2O_3: mp-19770; FeO: mp-18905).
